# Patient selection, ventricular tachycardia substrate delineation, and data transfer for stereotactic arrhythmia radioablation: a clinical consensus statement of the European Heart Rhythm Association of the European Society of Cardiology and the Heart Rhythm Society

**DOI:** 10.1093/europace/euae214

**Published:** 2024-08-23

**Authors:** Katja Zeppenfeld, Robert Rademaker, Amin Al-Ahmad, Corrado Carbucicchio, Christian De Chillou, Jakub Cvek, Micaela Ebert, Gordon Ho, Josef Kautzner, Pier Lambiase, Jose Luis Merino, Michael Lloyd, Satish Misra, Etienne Pruvot, John Sapp, Luis Schiappacasse, Marek Sramko, William G Stevenson, Paul C Zei, Dan Wichterle, Jonathan Chrispin, Claudia Herrera Siklody, Radek Neuwirth, Gemma Pelargonio, Tobias Reichlin, Clifford Robinson, Claudio Tondo

**Affiliations:** Department of Cardiology, Leiden University Medical Center, P.O. Box 9600, 2300 RC Leiden, The Netherlands; Department of Cardiology, Leiden University Medical Center, P.O. Box 9600, 2300 RC Leiden, The Netherlands; Electrophysiology, Texas Cardiac Arrhythmia Institute, Austin, TX, USA; Department of Arrhythmology, Centro Cardiologico Monzino IRCCS, Milan, Italy; CHU de Nancy, Cardiology, Institut Lorrain du Coeur et des Vaisseaux, Vandoeuvre Les Nancy, France; Radiation Oncology, University of Ostrava, Ostrava, Czech Republic; Electrophysiology, Heart Center Leipzig, Leipzig, Germany; Division of Cardiology, Section of Cardiac Electrophysiology, University of California San Diego, La Jolla, CA, USA; Cardiology, Institute for Clinical and Experimental Medicine, Prague, Czech Republic; Cardiology Department, University College London, London, UK; Cardiology, Hospital General La Paz, Madrid, Spain; Emory Electrophysiology, Electrophysiology Lab Director, EUH, Emory University Hospital, Atlanta, GA, USA; Atrium Health Sanger Heart Vascular Institute Kenilworth, Charlotte, NC, USA; Department of Cardiology, Lausanne University Hospital, CHUV, Lausanne, Switzerland; QEII Health Sciences Center, Halifax Infirmary Site, Halifax, NS, Canada; Department of Cardiology, Service de Radio-Oncologie, Lausanne University Hospital, CHUV, Lausanne, Switzerland; Cardiology, Institute for Clinical and Experimental Medicine, Prague, Czech Republic; Vanderbilt University Medical Center, Nashville, TN, USA; Professor of Medicine, Cardiac Electrophysiology, Brigham and Women's Hospital, Harvard Medical School, Boston, MA, USA; Cardiology, Institute for Clinical and Experimental Medicine, Prague, Czech Republic; Assistant Professor of Medicine, Johns Hopkins Medicine Division of Cardiology, Baltimore, MD, USA; Inselspital, Hôpital universitaire de Berne, Bern, Switzerland; Faculty of Medicine, Masaryk University, Brno, Czech Republic; AGEL Hospital Trinec-Podlesi, Trinec, Czech Republic; Cardiology Department, Policlinico Gemelli, Rome, Italy; Department of Cardiology, Inselspital, Bern University Hospital, University of Bern, Bern, Switzerland; Department of Radiation Oncology, Washington University School of Medicine, St. Louis, MO, USA; Department of Clinical Electrophysiology and Pacing, Monzino Cardiac Center, Milan, Italy

**Keywords:** Ventricular tachycardia, Stereotactic arrhythmia radioablation (STAR), Sudden death, Radiotherapy, Ablation

## Abstract

Stereotactic arrhythmia radioablation (STAR) is a novel, non-invasive, and promising treatment option for ventricular arrhythmias (VAs). It has been applied in highly selected patients mainly as bailout procedure, when (multiple) catheter ablations, together with anti-arrhythmic drugs, were unable to control the VAs. Despite the increasing clinical use, there is still limited knowledge of the acute and long-term response of normal and diseased myocardium to STAR. Acute toxicity appeared to be reasonably low, but potential late adverse effects may be underreported. Among published studies, the provided methodological information is often limited, and patient selection, target volume definition, methods for determination and transfer of target volume, and techniques for treatment planning and execution differ across studies, hampering the pooling of data and comparison across studies. In addition, STAR requires close and new collaboration between clinical electrophysiologists and radiation oncologists, which is facilitated by shared knowledge in each collaborator's area of expertise and a common language. This clinical consensus statement provides uniform definition of cardiac target volumes. It aims to provide advice in patient selection for STAR including aetiology-specific aspects and advice in optimal cardiac target volume identification based on available evidence. Safety concerns and the advice for acute and long-term monitoring including the importance of standardized reporting and follow-up are covered by this document. Areas of uncertainty are listed, which require high-quality, reliable pre-clinical and clinical evidence before the expansion of STAR beyond clinical scenarios in which proven therapies are ineffective or unavailable.

## Graphical Abstract

**Graphical Abstract:**
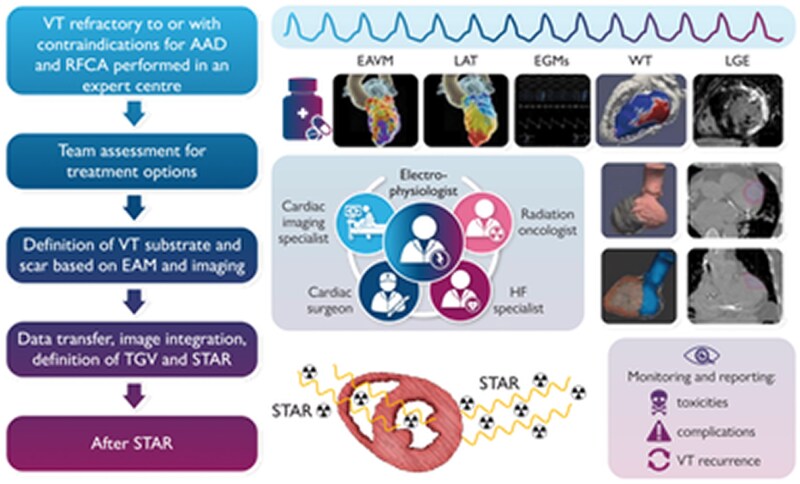

##  

1. Introduction
2. Cardiac stereotactic body radiation therapy: insights from animal models and clinical observations
 2.1. Animal studies
  2.1.1. Electrophysiological and histological response to cardiac stereotactic body radiation therapy
   2.1.1.1. Acute effects
   2.1.1.2. Early effects (2–6 weeks)
   2.1.1.3. Delayed effects (3–9 months)
  2.1.2. Adverse effects
 2.2. Human studies
  2.2.1. Characteristics of patients treated with stereotactic arrhythmia radioablation
  2.2.2. Time to effect
  2.2.3. Safety issues and complications
  2.2.4. Mortality
3. Stereotactic arrhythmia radioablation: technical aspects
 3.1. Target volume definition in radiation oncology
 3.2. Dose, doses on targets, organs at risk, and proposed constraints on organs at risk
 3.3. Target volume definition for stereotactic arrhythmia radioablation
4. Identification of the cardiac target volume
 4.1. Relation between ventricular tachycardia and myocardial scar
 4.2. Clinical or presumed clinical ventricular tachycardia
 4.3. Invasive mapping to identify scar and the ventricular tachycardia substrate
  4.3.1. Principles of electroanatomical mapping
  4.3.2. Voltage mapping
  4.3.3. Pace mapping
  4.3.4. Electrogram characteristics
  4.3.5. Functional substrate mapping
  4.3.6. Mapping during ventricular tachycardia
  4.3.7. Preparation for data transfer
 4.4. Non-invasive methods to identify scar and the ventricular tachycardia substrate
  4.4.1. Imaging
   4.4.1.1. Imaging of scar
   4.4.1.2. Imaging of ventricular tachycardia substrate
  4.4.2. Non-invasive definition and delineation of the ventricular tachycardia substrate by (body surface) electrocardiography
5. Patient selection for stereotactic arrhythmia radioablation
 5.1. Ischaemic cardiomyopathy
 5.2. Non-ischaemic heart disease
  5.2.1. Dilated cardiomyopathy
  5.2.2. Sarcoidosis
  5.2.3. Arrhythmogenic right ventricular cardiomyopathy
  5.2.4. Hypertrophic cardiomyopathy
6. Target volume transfer
 6.1. Image integration, data export, and inverse registration
  6.1.1. Electroanatomical mapping systems used for image integration
  6.1.2. Software for image registration
  6.1.3. Example of a workflow of image integration
 6.2. From the angio-computed tomography to the planning computed tomography
7. Future directions
Supplementary material
References

## 1. Introduction

Stereotactic arrhythmia radioablation (STAR), also previously referred to as stereotactic ablative body radiotherapy, is a new, non-invasive treatment for ventricular arrhythmias (VAs), which has been, after the first case report in 2015, applied in single cases, retrospective or small prospective series of highly selected patients with various substrates and different underlying etiologies. Early series have been promising; however, acute and long-term toxicity needs to be carefully evaluated and the medium to long-term effectiveness of arrhythmia suppression requires further study. Among published studies, the provided methodological information is often limited, and patient selection, target volume definition, methods for determination and transfer of target volume, and techniques for treatment planning and execution differ across studies, hampering the pooling of data and comparison across studies.^1^

Stereotactic arrhythmia radioablation requires close collaboration between clinical electrophysiologists, cardiac imaging specialists, and radiation oncologists, which is facilitated by shared knowledge in each collaborator's area of expertise and a common language. Based on a review of available published data and the personal experience of an international task force of physicians and scientists from centres with high-volume ventricular tachycardia (VT) ablation programmes, this clinical consensus statement aims to provide advice in patient selection and target volume determination for STAR. Technical aspects are summarized, and the personal experience with data transfer for treatment planning is provided.

To better understand the advantages and limitations of this emerging treatment, this group of experts suggests the use of uniform definitions related to cardiac target volumes and methodological information and outcome data to be provided in future scientific reports. It should be emphasized that this European Heart Rhythm Association clinical consensus statement is not intended as a guideline but as a support for clinical management.

The definitions of the category of advice and areas of uncertainty are provided in *Table 1*. The categories of supporting evidence and strength of evidence are listed in *Table 2*. Each statement listed in the table of advice has been discussed among the writing group members in an online meeting followed by a separate online voting.

**Table 1:** Definition of categories of advice and areas of uncertainty

Definition	Categories of advice	Icons
Evidence or general agreement that a given measure is clinically useful and appropriate	Advice TO DO	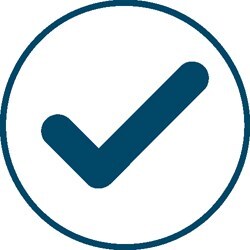
Evidence or general agreement that a given measure may be clinically useful and appropriate	May be appropriate TO DO	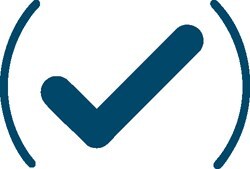
Evidence or general agreement that a given measure is not appropriate or is harmful	Advice NOT TO DO	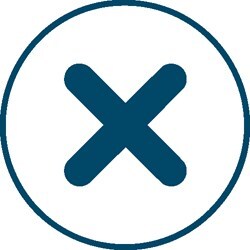
No advice can be given because of lack of data or inconsistency of data. The topic is important to be addressed.	Areas of uncertainty	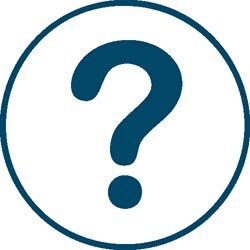

**Table 2:** Type and strength of supporting evidence

Type of supporting evidence	Criteria of evidence	Strength of evidence
Published data^a^	>1 high quality RCT Meta-analysis of high-quality RCT	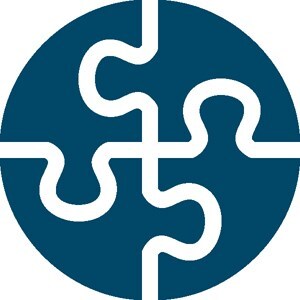
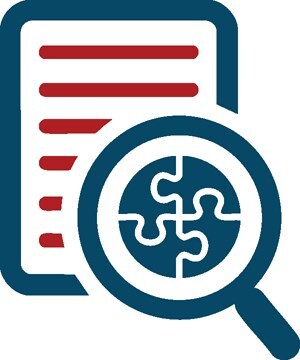	High-quality RCT > 1 moderate quality RCT Meta-analysis of moderate quality RCT	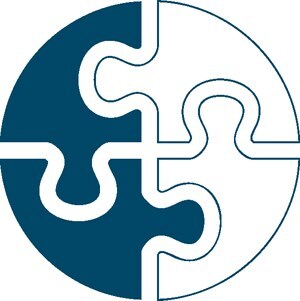
	High-quality, large observational studies	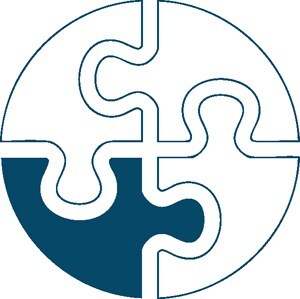
Expert opinion^b,c^	Strong consensus > 90% of WG supports advice	
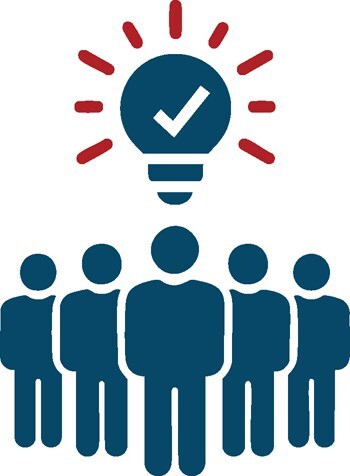	Consensus > 70% of WG supports advice	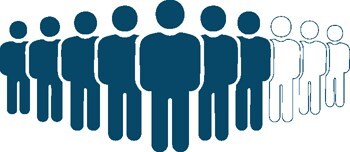

^a^The reference for the published data that fulfil the criteria is indicated in the table of advice, if applicable.

^b^Expert opinion also takes into account randomized, non-randomized, observational, or registry studies with limitations of design or execution, case series, meta-analyses of such studies, physiological, or mechanistic studies in human subjects.

^c^For areas of uncertainty, strong consensus/consensus that the topic is relevant and important to be addressed by future trials.

**Table:** Tables of advices

Patient selection, monitoring, and safety	Strength of evidence
**Advised TO DO**	
It is advised to consider STAR in the context of an approved investigational trial for patients with VT refractory to AAD (due to recurrence, intolerance, or contraindications) and RFCA performed in an expert centre.	
It is appropriate to discuss all patients considered for STAR with a multi-disciplinary team, including an electrophysiologist highly experienced in the invasive treatment of VA, a radiation oncologist, a heart failure specialist, a specialist in cardiac imaging, and a cardiac surgeon (for treatment alternatives and options in case of deterioration of cardiac function).	
It is advised to use standard reporting criteria for patient selection and patient follow-up.	
It is advised to capture and report all early and late recurrences of VA from the time of treatment, without any blanking period together with concomitant treatment in clinical trials.	
It is advised to systematically evaluate and report acute and long-term toxicities of STAR before the routine use of STAR.	
It is advised to evaluate radiation-induced toxicity according to at least the Common Terminology Criteria for Adverse Events (CTCAE) version 6.0.	
It is advised to perform regular clinical follow-up including a careful history of new or aggravated symptoms, ICD interrogation, and 12-lead ECGs to evaluate acute and long-term toxicities.	
It is advised to perform regular echocardiography for cardiac and valvular function, in particular if valves were in close proximity to the CardTV.	
**May be appropriate TO DO**	
STAR may be appropriate with appropriate institutional approval after failure of AAD treatment and catheter ablation by experienced operators that included available techniques to enhance lesion size and transmurality (e.g. half-saline, bipolar ablation, and TCEA) to define and reach the VT substrate.	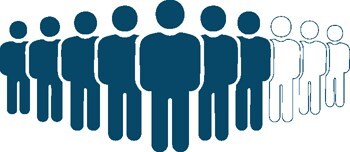
STAR may be appropriate if mechanical valves or LV thrombi preclude conventional catheter ablation provided that the VT substrate can be localized.	
It may be appropriate to perform cardiac ischaemia detection after 2–4 years if major coronary arteries were near and/or inside the CardTV.	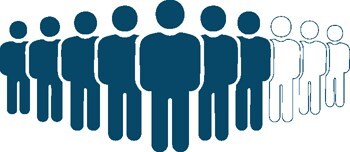
**Areas of uncertainty**	
It is unknown whether STAR is appropriate in patients with terminal end-stage heart failure whose dominant problem is heart failure arising from different aetiologies and not scar-related VT.	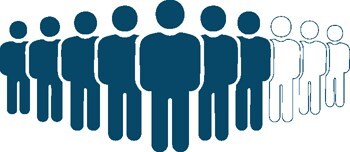
The role of STAR to *acutely* control recurrent VT or an ongoing ES is unclear.	
Optimal strategies to reduce acute and long-term side effects, apart from reducing radiation of organs at risk are unknown, e.g. the efficacy of PPI to reduce oesophageal or gastric toxicity is unknown.	
It is unknown if patients with interstitial lung disease are at higher risk, supporting a careful benefit-risk assessment.	

Technical aspects	Strength of evidence
**Advised TO DO**	
STAR for VA requires a standardized workflow allowing accurate integration of electrophysiological and anatomical data into planning by a multi-disciplinary team.	
It is advised to use uniform reporting criteria for constraints on OAR and measured dose on CardTV and OARs.	
It is advised to provide the technical details and choice of margins for the delineation of the planning target volume.	
**Areas of uncertainty**	
To date, there are no data suggesting the superiority of a specific SBRT system for STAR.	
The optimal dose, the exact response, and time to effect of STAR in diseased human myocardium of various aetiologies is unclear.	
Whether scar homogenization can be achieved by STAR is unknown.	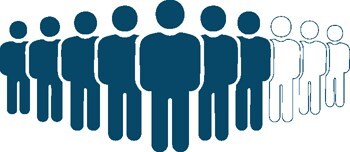
It is uncertain if patients benefit from STAR if dose constraints on OAR lead to dose reduction to the arrhythmia substrate.	

Definition and delineation of the target	Strength of evidence
**Advised TO DO**	
It is advised to use uniform definitions for CardTV in the context of STAR.	
It is advised to describe in detail the methods and criteria used to determine the CardTV.	
It is advised to incorporate invasive mapping data relevant to the arrhythmia for the determination of CardTV-EP until data are available showing reliability of the usage of only non-invasive methods such as ECGI.	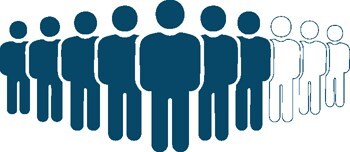
It is advised to specify the image modality and the applied methods and thresholds to define and delineate scar or fibrosis.	
For the electrophysiologist, for the CardTV, it is advised to include only safety margins that exclude areas with healthy functioning myocardium, as identified by voltage mapping or advanced CMR imaging (T1 mapping and LGE-CMR), and avoid unnecessary margins.	
For the radiation oncologist, it is advised to avoid unnecessary safety margins that include viable, functioning myocardium when planning the PTV.	
It is advised to obtain detailed EAM covering the surface of the chamber of interest with anatomical marking of at least 3 chambers/distinct landmarks in preparation of CardTV-EP_in_ whenever possible.	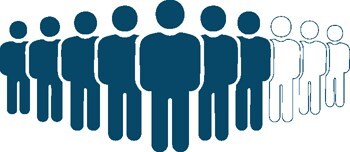
It is advised to indicate sites based on activation mapping, pace mapping, RF response, and functional information that are considered relevant by the EP on the electroanatomical mapping in patients who are considered candidates for STAR.	
**May be appropriate TO DO**	
It may be appropriate to exclude areas with BV >2.1 mV or BV 3.0 mV for remodelled or normal remote myocardium, respectively (recorded with 3.5 mm tip electrode) from CardTV-EP_inv_ in patients with ICM.	
It may be appropriate to deliver STAR to limited scar areas based on LGE-CMR in patients with prior MI.	
**Advised NOT TO DO**	
It is not advised to deliver STAR only based on LGE-CMR in patients with non-ischaemic fibrosis.	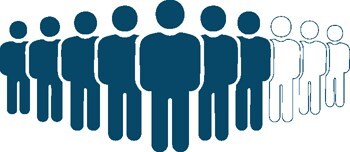
It is not advised to deliver STAR to an entire LV segment of the standard 17-segment LV model, if the CardTV-EP determined by mapping involves only part of that segment; irradiation of viable myocardium should be minimized.	
**Areas of uncertainty**	
It is unknown whether considering undocumented inducible VT morphologies for STAR treatment planning impacts outcomes.	
The reliability of ECGI only for defining the CardTV-EP is not known.	

## 2. Cardiac stereotactic body radiation therapy: insights from animal models and clinical observations

Stereotactic body radiation therapy (SBRT) is a well-established therapy to ablate solid tumours and some non-malignant targets throughout the body using targeted ionizing radiation.^2^ One important mechanism of tumour ablation is programmed cell death (in addition to apoptosis, probably also mitotic catastrophe and/or autophagy).^3^ The use of single fraction, high-dose ionizing radiation therapy (RT) via SBRT has recently been utilized as a treatment for patients with refractory VAs.^4,5^ The hypothesized anti-arrhythmic effect of STAR was initially presumed to be through a similar apoptotic mechanism. However, pre-clinical and clinical observations suggest a potentially more complex response of myocardium to STAR.

### 2.1. Animal studies

Pre-clinical studies were observational and investigated effects of various cardiac SBRT modalities in different animals and various experimental settings over medium-term follow-up (up to 8–10 months) (*Table 3*). Rabbit, mice, rat, pig, and dog hearts have been irradiated with various doses of carbon ions, protons, and photons.^6–10,16–19^ Most studies have been performed in healthy subjects or early after experimental myocardial infarction (MI).^9,16,17,19^ Cardiac SBRT targets included the pulmonary veins (PVs), the atrioventricular junction (AVJ), the cavo-tricuspid isthmus (CTI), and less frequently the myocardium of the left ventricle (LV). All doses have been delivered in a single fraction, unless stated otherwise.

**Table 3:** Animal studies

Animal model and energy	Treated cardiac structure	Dose	EP effects	Histological effects	Complications	Ref.
9 healthy mini pigs Photon beam	LA/PV	17.5–35 Gy (in 2.5 Gy step)	At 6 months: no PVI	At 6 month—around PV: 17.5 Gy no fibrosis, mild necrosis 20–27.5 Gy mild fibrosis, moderate necrosis 30 Gy moderate partial transmural fibrosis, mild necrosis 32.5 Gy intense but not transmural fibrosis, moderate necrosis 35 Gy most extensive damage, focal but mostly transmural fibrosis	At 6 months—35 Gy: focal fibrosis and fatty tissue necrosis with macrophages in the RA	^6^
85 Rats Photon beam	Whole heart	20, 25, 30, 40, 50 Gy	Within 1 month: AV conduction delay	Within 1 month: no myocardial necrosis and apoptosis; intra-cellular and extra-cellular oedema with diffuse vacuolization and intercalated disc widening		^7^
25 Healthy pigs Proton beam	AVJ; LV	AVJ: 30, 40, 55 Gy; LV: 30 GY at 2 sites; 40 Gy at 1 and 3 sites	AVJ: complete AV block at 10 and 12 weeks, or AV nodal conduction delay at 13 weeks LV: not reported	AVJ: heterogeneous lesions of mixed fibrotic changes and necrotic tissue at 3 months LV: homogeneous necrotic tissue in lesion core and necrotic mixed with haemorrhage in the lesion border at 12 weeks, dominant fibrotic tissue in lesion core at 40 weeks.	LV: severe vascular damage/MI (coronary artery included in the target volume at 30–40 Gy); pericardial effusion (non-specific inflammatory exudates) at 8–12 weeks, mostly improving afterwards; 6 sudden deaths	^8^
14 Swine Proton beam	LV post-MI scar	40 Gy; 30 Gy—2 targets (1 healthy tissue)	4 swine died suddenly due to monomorphic VT and/or VF	At 8 weeks: no significant changes in the infarct scar area. At 15 weeks: interstitial fibrosis in normal adjacent tissue and in the surviving myocyte regions. At 30 weeks: homogenized infarct area scar; myocytolysis with vacuolar degeneration and fibrosis in normal tissue both in swine treated with 30 and with 40 Gy	In all swine evidence of pericardial effusion (maximum at 8 or 12 weeks, improving afterwards) (sterile fluid with small number of neutrophils) 4 swine died suddenly of VF	^9^
14 Pigs Heavy ion beam	AVJ; PV-LA junction; LV freewall	AVJ: 25, 40, 55 Gy; RSVP-LA junction LV-free wall: 40 Gy	AVJ at 6 months: 25 Gy no effects; 40 Gy transient AV block; 50 Gy complete AV block RSVP-LA at 6 months: decreased voltage amplitude. LV at 3 months: no significant decreased voltage amplitude; at 6 months decreased voltage amplitude	AVJ: transmural fibrosis, cardiomyocyte disarray and haemorrhage in case of complete AV block. At 3 months: prevalence of haemorrhage and inflammation. At 6 months: prevalence of fibrosis		^10,11^
4 explanted pig hearts Heavy ion beam	AV-node	70–90–160 Gy	Immediate effect: 70 Gy: no acute conduction disturbance 90 Gy: AV prolongation in heart with previous 2nd-degree AV block 160 Gy: total AV block in all hearts	Analysed immediately after radiation For 160 Gy: no macroscopic damage, no apoptosis or necrosis. No increased expression of apoptosis protein markers. Phosphorylated histone 2AX (DNA damage marker) was strongly positive) Hyper eosinophilia was noted in the area.	N.A.	^12^
10 mini pigs B-radiation through catheter	Pulmonary veins	60 Gy	Acutely: No PV conduction block seen as treatment delivery was segmental and PVs are circumferential	Immediately after ablation: PV sleeve necrosis and lamina disruption After mean 81 ± 27 days: mild focal neointimal hyperplasia and PV necrosis	N.A.	^13,14^
16 mini swine Gamma-radiation	CTI AV-node PV LAA	25–80 Gy	Time to evaluation: 25–196 days CTI: decreased voltage and block LAA: decreased voltage PV: decreased voltage AV node: AV block at 49 days	After range 25–89 days: transmural loss of myocyte architecture and increased fibrin, inflammatory changes (monocyte infiltration) and discrete areas of transmural fibrosis.	None	^15^
Non transmural ventricular MI in 24 rabbits Heavy ion	LV	15 Gy	2 weeks after radiation: Reduced inducibility by PES for VT/VF in radiated hearts	After 2 weeks: no fibrosis, but an increase in connexin-43	N.A.	^16^
25 pigs Proton beam	AV junction (5) LV (20)	30–40–50 Gy	N.A.	At 12 weeks: first necrotic tissue at core and fibrosis at border. At 16–24–40 weeks, fibrosis became predominant.	N.A.	^8^
8 mini pigs Gamma radiation	Upper right PV	22.5–40 Gy	6 months post-radiation: no PV isolation achieved	6 months post-ablation: 22.5 Gy: fatty tissue necrosis >30 Gy: fibrosis was seen >40 Gy: complete AV block secondary to AV node fibrosis	At 37.5 Gy, a fatal bronchomediastinal fistula was seen.	^14^

AV, atrioventricular; AVJ, atrioventricular junction; CTI, cavo-tricuspid isthmus; EP, electrophysiological; LA, left atrium; LAA, left atrial appendage; LV: left ventricle; MI, myocardial infarction; PV, pulmonary vein; PVI, pulmonary vein isolation; RA, right atrium; RSVP, right superior pulmonary vein; VF, ventricular fibrillation; VT ventricular tachycardia.

#### 2.1.1. Electrophysiological and histological response to cardiac stereotactic body radiation therapy

Time- and dose-dependent functional and morphological changes have been reported.^6–10,16–19^

##### 2.1.1.1. Acute effects

There are limited animal data on the acute effect of cardiac irradiation. Acute conduction delay/block of the AV junction could only be achieved with ≥90–160 Gy irradiation, which is much higher than the clinically applied dosage of 20–25 Gy in humans.^10^

##### 2.1.1.2. Early effects (2–6 weeks)

Pulmonary vein muscular sleeve necrosis and lamina disruption have been described after 60 Gy of beta-radiation of PV in healthy swine.^13^ In a rabbit infarct model, heavy ion irradiation 2 weeks after infarction resulted in up-regulation of Cx43 observed 2 weeks after irradiation in the infarct border zone (BZ) and remote myocardium.^16^ Inducibility of VT/ventricular fibrillation was reduced compared with infarcted non-radiated animals.^16^ In a murine infarct model, gamma-radiation 2 weeks after infarction increased conduction velocities in normal tissue and in the infarct BZ 6 weeks after radiation, with an increase in voltage-gated sodium channels and Cx43 proteins, which would be likely to improve cell coupling and tissue conduction velocities. These changes were observed at doses as low as 15 Gy. In healthy rabbit hearts but not in the murine infarct model, up-regulation of Cx43 was demonstrated up to 1 year.^16,19^ After high-dose irradiation *of whole rat hearts* with 20–50 Gy,^7,20^ intra-cellular and extra-cellular oedema with diffuse vacuolization and intercalated disc widening has been observed with a more pronounced effect after ≥30 Gy occurring within the first month and without evidence of necrosis. The early changes coincided with PR and QTc prolongation. These data suggest different mechanisms that may contribute to early modification of conduction. A mini swine study of focused X-beam radiation with dose escalation (25–50 Gy CTI, 40–70 Gy AV node) and repeated electroanatomical mapping (EAM) of target structures after 25–196 days showed decreased voltages at all doses. The authors concluded that a dose of 25 Gy or larger is needed to create a lesion that alters electroanatomical characteristics. Of note, bidirectional CTI block was only observed at 30 days and 40 Gy and complete AV block at 49 days and 70 Gy.^15^

##### 2.1.1.3. Delayed effects (3–9 months)

The applied doses seemed to correlate with observed histopathological effects and the degree of fibrosis during follow-up.^6,10^ Intimal PV high-dose beta-radiation with 60 Gy at 1 mm depth in pigs resulted in acute endothelial denudation, disruption of elastic lamina, and myocardial sleeve necrosis followed by fibrosis after 81 ± 27 days.^13^ In general, apoptosis and necrosis followed by fibrosis are more likely to explain delayed effects. Within the first month, no myocardial necrosis or apoptosis was reported.^7,16^ Endothelial microvascular damage and signs of haemorrhage and inflammation were followed by various degrees of fatty tissue and myocardial necrosis, with progressive fibrosis formation over the medium term. In a dose-finding study, fibrosis was seen following gamma irradiation of the porcine atrial PV junction with doses above 30 Gy.^14^ Others reported mild fibrosis 6 months after gamma irradiation with 20–27.5 Gy.^17^ Lesion stabilization has been described within a variable time interval, typically between 3 and 9 months.^6–11,19,20^ Following proton beam radiation (40–50 Gy) of the porcine AVJ monitored by implanted pacemakers, significant conduction delay was observed after 10–12 weeks with heterogeneous lesions of mixed fibrosis and necrotic tissue at 3 months.^8^

#### 2.1.2. Adverse effects

In porcine models, radiation dose has been correlated with cardiac toxicity. Relatively low doses of radiation (<35 Gy) resulted in no or only mild cardiac or extra-cardiac toxicity with minor segmental or global systolic dysfunction and small pericardial effusions.^8^ Higher doses (>35 Gy) were associated with severe cardiovascular damage, acute arrhythmogenicity (VAs and sudden death), and extra-cardiac effects. A bronchomediastinal fistula was reported after administration of 37.5 Gy.^14^ Acute vascular damage and occlusions were reported in one study where proton doses of up to 40 Gy were specifically delivered to the coronary arteries.^8^

### 2.2. Human studies

Since the publication of the first two case reports on STAR in patients with VT refractory to available therapy in 2014/2015,^21,22^ the number of reported cases, case series, and small prospective series has increased significantly (*Table 4*). The first series of five patients with ischaemic cardiomyopathy (ICM) and non-ischaemic cardiomyopathy (NICM) was published in 2017, and the largest series to date included 36 patients treated in Czech Republic since 2014.^39^ In all reports, a single fraction of 25 Gy has been applied.

**Table 4:** Human studies on STAR for ventricular tachycardia

Study	No. of patients	LVEF, % mean or *median*	VT burden pre- STAR	Indication for STAR	(Prior) mapping approach	Follow-up	VT recurrence	Blanking period post-STAR	VT burden post- STAR	AAD-use pre- and post-STAR	Mortality and complications
*Cuculich* ^5^	5 (2 ICM, 3 NICM)	23	Median 1315 [5–4312] episodes within 3 months	3 pts previous RFCA failure 1 frail pt 1 pt mechanical valve	Non-invasive EAM 3/4 prior invasive EAM	Median 12 months	4/5 (80%)	6 weeks	99.9% reduction in VT burden, ICD ATP/shocks 1 patient underwent re-RFCA	Amio: 100% vs. 25% Mexiletine: 100% vs. 0%	20% (1/5) Fatal stroke 3 weeks after STAR,
*Robinson* ^23^	17 (11 ICM, 6 NICM) (+2 pts with PVC)	*25*	Median 119 [4–292] episodes within 6 months	3 pts mechanical valves 1 frail patient, 1 LV thrombus	Non-invasive EAM 12/17 prior invasive EAM	Median 13 months	11/17 (65%)	6 weeks	Median 3 [0–31] episodes	At 6 months: Amio: 59% vs. 47% Class I: 65% vs. 12% Sotalol: 41% vs. 35%	35% (6/17) mortality after 12 months of complications: 1 possible HF, 1 possible pericarditis, 2 pneumonitis requiring therapy
*Neuwirth* ^4^	10 (8 ICM, 2 NICM)	27	Average 18 episodes within 3 months	Inaccessible substrate during RFCA, bailout procedure	All endo (median 2 procedures), epi in 9/10	28 [16–54] months	8/10 (80%)	90 days	Average 0.93 episodes, two patients with higher VT burden, one patient re-RFCA	Amio: N.A. vs. 30%	30% (3/10) mortality Possible increase in mitral regurgitation post-SRT
Lloyd^24^	10 (4 ICM, 6 NICM)	N.A.	20.1 ATP/shocks per month pre treatment	Inaccessible substrate during RFCA, bailout procedure	All endo, 4 epi, 1 epi surgical	Median 5 months	8/10 (80%)	No blanking period	48% reduction in ATP, 68% reduction in shocks	Amio: 100% vs. 100% Class I: 80% vs. 80% Other: 30% vs. 30%	5/10 (50%) patients received HTx or were sent to palliative care 2 patients with pneumonitis
Abdel-Kafi^25^	2 (2 NICM)	36	Incessant VT	>3 failed RFCA, bailout procedure	All endo and epi	Average 7.5 months	1/2 (50%)	No blanking period	1 slow VT terminated by ATP	N.A.	None
*Gianni* ^26^	5 (4 ICM, 1 NICM)	34	Average 101 episodes within prior 12 months	Inaccessible substrate during RFCA, bailout procedure	All endo, 2/5 epi	12 ± 2 months	5/5 (100%)	No blanking period	All patients had clinically significant VT recurrences requiring RFCA or AAD	Amio: 100% vs. 100%, 3 patients with AAD escalation	40% (2/5) patients died due to HF No complications reported
Chin^27^	8 (4 ICM, 4 NICM)	21	Electrical storm in 6/8, 2 patients with >50 episodes within 12 months prior to STAR	6 pts failed multiple RFCA (endo/epi) 1 pt refused RFCA 1 frail pt	6/8 patients with mean 1.6 ± 1.6 ablations, 4/8 epi. 2 patients without prior RFCA	Median 7.7 [5–10 months	3/5 (60%),	No blanking period	No change in ICD therapies prior/post-STAR ES patients: time to effect not specified	Amio: 75% vs. 38% Class I: 63% vs. 63% 2 patients stopped amio due to toxicity	38% (3/8) patients died within 3 months No complications reported
*Ho* ^28^	6 (2 ICM, 4 NICM)	29	Electrical storm in 5/6	Bailout after failing all available strategies including RFCA	Mean 2.2 ± 1.0 prior RFCA, all endo	Mean 6.0 ± 4.9 months	2/6 (33%)	6 weeks	0.67 ± 1.0 shocks per patient	Amio: 100% vs. 100%	17% (1/6) patients died No complications reported
Ho^29^	6 (1 ICM, 5 NICM)	45	Average 15 VT episodes in 3 months prior to STAR	Recurrent VT or contraindication to RFCA, 1 pt LV thrombus	5/6 endo, 3/6 ≥ 2 RFCA	Median 15 [10–20] months	4/6 (67%)	6 weeks	Average of 4.6 VT episodes during follow-up	All AAD: 100% vs. 100%	33% (2/6) patients died 1 patient with PE requiring intervention
Lee^30^	7 (5 ICM, 2 NICM)	27	Around average 66 episodes per patient	Prior failed RFCA or contraindications (severe HF and patient frailty)	Median 2 [0–3) endo, STAR based on NI-EAM	Average 4.6 months	4/5 (80%)	No blanking period	85% VT suppression, 1 patient underwent re-RFCA	Amio: 86% vs. 43% Mexiletine: 14% vs. 14%	43% (3/7) patients died within 4 weeks (worsening HF), no (other) complications reported
*Carbucicchio* ^31^	7 (3 ICM, 4 NICM)	27	Electrical storm in 4/7 patients, mean 29 ± 33 VT episodes at 3 months prior to STAR	Contraindication to RFCA or otherwise not suitable for interventional approach 3 pts mechanical valves 2 pts LV thrombus	Endo 5/8, epi in 2/8, non-invasive (only CT and ECG) in 3/5	Median 8 months	3/4 (75%)	6 weeks	For 4 patients: mean 2 ± 2 VT episodes at 6 months. ES patients: time to effect not specified	Amio: 100% vs. 100%	43% (3/7) patients died within 3 months
Qian^32^	6 (6 ICM)	*20*	All electrical storm	At least 1 RFCA attempt and substrate deemed unreachable or procedural risks were deemed too great	All endo, median 2 [2–4] procedures, 2/6 epi, 3 needle/alcohol ablation	Median 8 [7–10] months	5/6 (83%)	No blanking period	5/6 patients with VT recurrence, with median 97 days [IQR 73–97}, 4/6 with ICD shock. No change in ICD VT readout 6 months pre-/post-STAR. Repeat RFCA in 4/6 patients	All AAD: 100% vs. N.A.	50% (3/6) patients died due to HF Complications: 1 pneumonia, 1 LVEF deterioration
Gerard^33^	2 (2 ICM)	32	30 and 22 episodes prior to **STAR**	1 LV thrombus 1 patient deemed too complex due to multiple VT morphologies at previous RFCA	1/2 patients underwent 1 RFCA, the other substrate was based on 4D VT and ECG	Mean 14.5 months	1/2 (50%)	No blanking period	Patient without recurrence had no VT in 1 month prior to STAR, other patient no difference in ICD therapy	Amio: 100% vs. 100%	0%, no complications reported
*Molon* ^34^	6 (4 ICM, 2 NICM)	30	All electrical storm	1 mechanical valve, others unclear; unfitness/failure of RFCA or AAD resistant VT. No specifications.	Non-invasive mapping	Median 13 months	3/6 (50%)	No blanking period	2/6 patients VT recurrence at 2 and 5 months, other patient N.A.	N.A.	17% (1/6) patients died 1 month post-STAR
Ninni^35^	17 (10 ICM, 7 NICM)	34	ES in 14 pts (71%) Incessant VT in 5 (29%) Median 20 [12–61] within 1y; 8 [4–21] within 1 month before STAR	Procedural failure after RFCA in 14, 1 patient without proper vascular access, 2 LV thrombus	Endo in 14 patients, epi in 2 patients, 3 other patients based on MRI and CT	Median 13 [11–18] months	10/17 (59%)	6 weeks	1 patient underwent re-RFCA, reduction in ATP and shocks. 59% (10/17) at least 1 treated VT after follow-up Incessant VT patients: 4/5 ‘major’ decrease in VT burden	Amio 41% vs100%	24% (4/17) patients died, 2 within 6 weeks, 1 after 6 months, 1 after 13 months No complications requiring intervention reported
*Aras* ^36^	8 (2 ICM, 6 NICM)	24	Median 42 episodes, range [3453–22], average ICD shocks 8.46/month	Failed RFCA in 4 patients, 3 pts with mechanical valves 1 pt with LV thrombus	Endo in 5 patients, epi in 4, 3 others based on MRI	Median 8 [range, 1–14] months	8/8 (100%)	No blanking period	At 12 months post-STAR, 5 patients had ≥2 ICD shocks All patients showed ‘significant’ VT reduction within 2 weeks of STAR	Amio: 88% vs. N.A. Mexiletine: 50% vs. N.A.	50% (4/8) patients died 1 patient had PE requiring treatment
*van der Ree* ^37^	6 (6 ICM)	*38*	Median 31 [8–138] treated VT episodes before STAR	At least one RFCA and AAD failure or contra-indications for RFCA	6/6 patients with median of 2 [1–5] RFCA, 2 epi STAR based on NI-EAM	Median 12 months [7–12]	5/6 (83%)	6 weeks	Median 9 [0–109] treated VT episodes during follow-up	Amio 83% vs. 83% Mexiletine: 83% vs. 83%	33% (2/6) patients died 1 pt had PE requiring treatment, 2 pts had LVEF deterioration
*Herrera* ^38^	20 (6 ICM, 9 NICM, 4, inflammatory 1 metastasis)	31	8 cases with electrical storm. Median 108 (3–5502) episodes 6 months before	Refractory to conventional treatment. For patients where the critical part of substrate was inaccessible.	At least one RFCA with endo and/or epi map. Except for patient with metastasis and one with LV thrombus. Ethanol infusion was attempted if conceivable	Median 25 (3–48) months	18/19 (95%) of patients who were alive 1 week after STAR	No blanking period	Median of 10 (0–150) events 6 months after STAR. In the nine patients with an ES, eight (89%) were successfully controlled by STAR Twelve (67%) of the 18 patients with VT recurrence underwent a redo RFCA procedure. A median of 1 (1–5) redo RFCA was performed, the first one at a median of 9 months after STAR: 5 for ES, 1 for incessant slow VT and 6 for recurrent fast VT	STAR allowed tapering of the prescribed dose of amiodarone in 4 (36%) out of 11 patients.	7/20 (35%) patients died. 1 patient had electrical storm, 1 pericardial fibrosis, 1 rib fracture, 1 fast progression to severe aortic stenosis
**Total**	**148** **80 ICM** **63 NICM** **4 inflammatory** **1 metastasis**		**56 (38%) ES or incessant VT**	**11 (7%) mechanical valves** **9 (6%) thrombus** **66 (45%%) bailout/inaccessible substrate** **62 (42%) not specified (e.g. frailty patient, complex substrate, contra-indications for RFCA)**	**79% (96/122) of specified patients had invasive conventional EAM**		**85/128 (66%)**			**8/18 same** **3/18 reduction** **2/18 increase** **5/18 not specified**	**Mortality:** **34% (50/148)**

Studies indicated by italic font first authors name have included patients prospectively.

AAD anti-arrhythmic drugs; ATP, anti-tachycardia pacinganti-tachy pacing; EAM electro-anatomical map; ECG, electrocardiogram; ES, electrical storm; HF, heart failure; HTx, heart transplant; ICD, implantable cardiac defibrillator; ICM, ischaemic cardiomyopathy; LV, left ventricular; LVEF, left ventricular ejection fraction; MRI, magnetic resonance imaging; NI, non-invasive; NICM, non-ischaemic cardiomyopathy; PE, pulmonary embolism; RFCA, radiofrequency catheter ablation; STAR, stereotactic arrhythmia radioablation; VT, ventricular tachycardia.

#### 2.2.1. Characteristics of patients treated with stereotactic arrhythmia radioablation

The majority of patients considered for STAR had prior failure of at least one radiofrequency catheter ablation (RFCA) procedure, usually with appropriate attempts to reach the VT substrate [combined endo/epicardial access and/or endocardial right ventricle (RV) and LV, often including bailout strategies]. Other indications have included mechanical valves or LV thrombus, impeding or excluding conventional RFCA, prior cardiac surgery hampering percutaneous epicardial access, and occasionally patient frailty. Treated patients have had a variety of structural heart diseases (SHDs), with series reporting median ages between 60 and 70 years, although patients as young as 23 years and older than 81 years have been included as well. In comparison with reported cohorts treated with catheter ablation (CA), the reported LV ejection fraction (LVEF) at the time of treatment has been similar (20–45%); however, the number of patients in New York Heart Association functional Class III/IV has been higher in patients undergoing STAR, which may be partly explained by a higher threshold to apply unconventional therapy and thus more severe arrhythmic presentation with a high VT burden, frequent implantable cardiac defibrillator (ICD) shocks, and use of combinations of anti-arrhythmic drugs (AADs). While the majority of treated patients had a high burden of sustained monomorphic VT, three patients underwent STAR for a high premature ventricular complex (PVC) burden after the failure of RFCA^23,28,35^ with a reported reduction in PVC burden. It is, however, unclear if the effect of STAR on an automatic focus is the same as on a re-entrant circuit.

#### 2.2.2. Time to effect

The interpretation of acute effects of STAR can be difficult due to the multiple treatments for heart failure and arrhythmias often applied during the same period in this population.^25,35,40^ A case of acute termination of VT^30^ and a decrease in duration of VT runs within 48 h of STAR have been reported.^41^ More often, a significant reduction of VT burden has been reported 1–7 weeks after radiation,^24,28,30,35,36^ and the full effect may not occur for several months.

The time to effect is an important consideration for patient selection. In patients with an ongoing electrical storm, STAR cannot be relied on to *acutely* terminate the arrhythmia causing the storm. Recognition of the delayed effect also influences the selection and timing of other therapies, such as repeat RFCA, cardiac sympathectomy, and AADs for continued or recurrent arrhythmias after ablation. The optimal approach regarding AAD after STAR is currently unknown. In some series, a reduction in VT burden and ICD therapies after STAR has allowed for AAD tapering. However, VT recurrence and reduction of ICD therapies vary across cohorts. In two case series, including mainly patients with severely depressed LV function, VT recurred in all, with no change in ICD therapies after STAR.^27,36^ In 8 of 17 series, AAD treatment was continued, in 2 AAD, therapy was escalated after STAR, and in 5, no data were provided. Reports of long-term outcomes are also limited. Late recurrence of VT, 6–12 months after STAR, has been reported. Preliminary observations suggest that STAR may be safely repeated in some patients, but more study is needed.^42–44^ In particular, data are needed regarding early and late recurrences, together with concomitant treatment in clinical trials to get more insights into efficacy and the time to effect.

#### 2.2.3. Safety issues and complications

Before widespread clinical implementation of STAR for VA, acute and long-term toxicities should be carefully evaluated and thoughtfully considered. Until now, direct radiation of cardiac structures by oncologists has been avoided because of potential toxicity.^45,46^ The American Association of Physicists in Medicine Task Group Report TG101 and others have recommended that the heart volume receiving ≥16 Gy (V16) should be kept below 15 cc and that the max heart dose should be less than 22 Gy.^47,48^ STAR for VA aims to intentionally irradiate myocardium. Although the myocardial treatment area usually contains scar tissue, the risk of damage to adjacent cardiac structures including contractile myocardium contributing to function, valves, coronary arteries, and non-cardiac organs at risk (OAR) is of concern. In the reported series, acute toxicity appears to be reasonably low, but long-term toxicity remains unknown and events can occur even many months or years after STAR. Available data on adverse events following cardiac STAR for VT are provided in *Table 4*.

Acute toxicity includes nausea, pericarditis, esophagitis, or pneumonitis. Long-term toxicities include obstructive coronary artery disease (CAD), aortic or mitral stenosis or regurgitation, pericardial effusions, and constrictive pericarditis.^49^ Significant progression of pre-existing mitral regurgitation after STAR has been observed in 25% of 32 cases, and three (9%) patients had to undergo mitral valve intervention (Grade 4 toxicity)^39^ In a series of 20 patients with mildly reduced LVEF (median LVEF 46%) and a planning target volume (PTV) of 25 cm^3^ (18–39 cm^3^) and a median whole heart dose of 6.1 Gy, there was no significant reduction in LVEF [median before: 38%, range (24–52), relative change +12%, range (−26% to +19%)] but worsening of valve function (≥2 grades) occurred in 25% of patients after a median of 1.1 years.^50^ Occurrence of significant CAD 2–3 years after STAR may justify routine cardiac ischaemia detection, if coronary arteries are close to the PTV. In patients treated for lung cancer, the risk of radiation-induced lung toxicity, including radiation pneumonitis and pulmonary fibrosis, is associated with the dose and irradiated volume of lung tissue. Patients with pre-existing interstitial lung disease appeared to be especially susceptible to severe adverse effects.^51^ Following STAR, radiation-induced pneumonitis has been reported in case series systematically performing a computed tomography (CT) scan at 3 months. The prognosis was generally good, although some cases required limited steroid treatment.^5,23^ Dose constraints also apply to the upper gastrointestinal tract,^52^ and proximity to the stomach often limits STAR target coverage in pancreatic, liver, or adrenal tumours.^53^ Similar dose optimization might be necessary in STAR, particularly when the VT substrate is located at the inferior wall. However, robust clinical estimates of the individual risk are limited. Toxicity may be influenced by other factors such as gastric filling.^54^ Two cases of STAR-related esophagitis (10%) were reported with one related death (Grade 5 toxicity) due to unresectable oesophago-pericardial fistula 9 months after STAR.^55^ A case of a gastro-pericardial fistula requiring surgical repair was also observed in the ENCORE VT cohort.^23^ Prophylactic medication to minimize oesophageal luminal acid after thermal ablation for atrial fibrillation is commonly used in practice. However, data supporting the benefit of this strategy are lacking, and the benefit of this strategy in patients undergoing STAR is similarly unclear.

Since most of the previously published series reported on adverse effects within relatively short follow-up (mostly up to 1 year), the occurrence of late adverse effects may be under-reported. We advise evaluation of radiation-induced toxicity according to the Common Terminology Criteria for Adverse Events. Patients should be closely followed to monitor not only VA recurrences by serial ICD interrogations or remote monitoring but also treatment-related acute and long-term toxicity. Electrocardiogram (ECG), echocardiography for cardiac and valve function, and further diagnostic evaluation in case of any symptoms suspicious for pneumonitis or pulmonary fibrosis or digestive symptoms suggestive of organ toxicity are advised.

#### 2.2.4. Mortality

Despite the reduction in VT burden and ICD therapies, reported death rates are high; 34% of patients included in the case series died during a median follow-up of ∼12 months (*Table 4*). In a series of 36 patients,^39^ 50% died after a median follow-up of 26.9 months. In a pooled analysis of observational studies, 1-year mortality was 32% (95% confidence interval 23–41), with almost half of the deaths occurring within 3 months after treatment.^56^ Death was often attributed to rapidly progressive heart failure and considered unrelated to STAR. The high mortality may be due to advanced underlying disease or comorbidities, but an adverse effect of STAR cannot be excluded with certainty. As a consequence, patient selection for STAR ideally requires a team composed of an electrophysiologist experienced in the invasive management of VA, a radiation oncologist, a heart failure specialist, a specialist in cardiac imaging, and, in some cases, a cardiac surgeon to consider treatment alternatives and options in case of deterioration of cardiac function after irradiation. Randomized trials will be needed to determine the risk-benefit ratio of STAR.

## 3. Stereotactic arrhythmia radioablation: technical aspects

Stereotactic arrhythmia radioablation has been performed using existing treatment systems to deliver ionizing radiation to arrhythmia substrates. Well-established principles in the field of radiation oncology have been adapted for STAR, particularly relating to mechanism of energy delivery and motion management. To date, there are no data suggesting different outcomes between different treatment systems, although head-to-head comparisons are lacking. *Table 5* summarizes important aspects of the two main treatment planning and delivery platforms applied in STAR.

**Table 5:** Different available systems

System 1: Isocentric C-arm linear accelerator with X-ray or MRI guidance (LINAC)		
Methods and characteristics of dose delivery and verification imaging	Motion management approaches (respiratory motion)	Delivery guidance approaches
Beam gating for beam on/off control to minimize treated volume due to respiratory motion High-dose rate and fast dose delivery (using FFF beams) High conformity, steep dose gradient, and fast delivery of high dose (using IMRT, IMAT, VMAT, and MLC) 6D treatment couch for compensation of patient's position and orientation detected during verification imaging	*Treatment at free breathing* ITV concept to cover all predicted target positions based on simulation imaging (e.g. 4DCT and multiple single-phase CT) ITV concept with application of abdominal press to reduce respiratory motion range decreasing treated volume but keeping ‘free‘ breathing Beam gating at selected phase of respiratory cycle (e.g. expiration) controlled by respiratory phase monitor with target defined and treatment plan optimized on same-phase simulation CT acquired using same respiratory phase monitor *Treatment at breath-hold* Beam gating at selected breath hold controlled by respiratory phase monitor with target defined and treatment plan optimized on same-phase simulation CT	Pre-treatment (repeated during treatment in addition) non-gated 3D CT Pre-treatment (repeated during treatment in addition) gated 3D CT or 4D CT registered to same-phase reference planning CT 3D MRI to match with reference MRI planning images with 2D single-slice MRI of liver dome at breath hold for beam gating
Treatment planning based on: X-ray 3D/4D CT (cone beam CT) 3D MRI		

System 2: Stereotactic robotic system CyberKnife		
Methods and characteristics of dose delivery and verification imaging	Motion management approaches	Delivery guidance approaches
High-dose rate and fast dose delivery (using FFF beams) Robotic technology for maximum flexibility of beam directions for high conformity and gradient of dose distributions Three types of secondary collimator (including extra-small apertures) for high conformity and gradient of dose distributions Integrated compensation of spatial deviations, including patient orientation and 3D position of target Target tracking (by treatment beams) based on correlation model between target position and signal of LEDs placed on patient's chest to minimize target positional uncertainty and treated volume due to respiratory motion at free breathing	*Treatment at free breathing* Target tracking using: ICD lead Temporary pacing lead Fiducial placed before therapy delivery	Pre-treatment 6D correction of patient orientation based on pair of X-ray images auto-registered to DRRs On-treatment beam position and direction corrections and tracking based on continuous monitoring of target surrogate 3D position detected from X-ray images correlated with signal of external respiratory monitor

6D, degrees of freedom; FFF, flattening filter free; DRR, digitally reconstructed radiographs; ECG, electrocardio graph; ICD, implantable cardioverter defibrillator; IMAT, intensity-modulated arc therapy; IMRT, intensity-modulated radiotherapy; ITV, internal target volume; MLC, multi-leaf collimator; MRI, magnetic resonance imaging; VMAT, volumetric arc therapy.

In order to minimize the risk of non-targeted tissue toxicity, the conformation of high doses to the target and rapid fall-off doses away from the target is critical. The process requires delineation of the target volume, incorporation of motion management strategies, and dose constraints for OAR. Accordingly, complex pre-treatment imaging and precise image-guided radiotherapy (IGRT) are crucial prerequisites for STAR.

### 3.1. Target volume definition in radiation oncology

Available information is increasing on the specific electrophysiological and imaging findings used to define target volumes for STAR, as well as on technical aspects related to radiotherapy such as the prescribed dose, the treated volumes, and the doses received by these volumes including the OAR. However, across available series, there is a large heterogeneity regarding volume definitions, methodologies applied, and quality of provided procedural and technical data. *Table 6* provides an overview of volume definitions used in oncology.

**Table 6:** Overview of existing definitions in radiation oncology

Definition in (radio-) oncology	Definition	No. of STAR studies reporting the volume	Volumes treated (if provided)	Remarks relevant for STAR
Gross target volume **(GTV)**	The demonstrable extent of tumour extension and location. GTV and target volume (TV) can be used interchangeable.	7	Median 25.1 mL Range 6.4–88.6 mL	To date, no image or mapping technique can accurately delineate the extent of VT substrates.
Clinical target volume **(CTV)**	Considers a volume of tissue containing subclinical malignant disease, with a certain probability of occurrence relevant to the selected therapy.	5	Median 34.6 mL Range 6.75–108.7 mL	For STAR, there is no equivalent. It is advised to avoid any functioning myocardium.
Internal target volume **(ITV)**	CTV plus a safety margin that considers uncertainties in the size, shape, and position of the CTV within the patient.	12	N.A.	It is advised to use approaches with advanced motion management to minimize the ITV.
Planning target volume **(PTV)**	A geometric concept introduced for treatment planning and evaluation. In the ICRU definition, the PTV is a margin around the CTV, taking into account the patient's internal and setup uncertainties.	11	Median 81.2 Range 3.5–345.1 mL	For STAR different margins ranging between 5 and 10 mm have been used.

### 3.2. Dose, doses on targets, organs at risk, and proposed constraints on organs at risk

The average prescribed dose in prior reports was 24.93 Gy [median 25 Gy (range 15–27.6 Gy)].^38^ The decision to treat one patient with 15 Gy was based on the need to reduce the dose to respect the constraints on OAR,^27^ thereby prioritizing OAR constraints over target coverage. Constraints on OAR are based on prediction of normal tissue toxicity resulting in a restriction on the doses to individuals. In all but one published case, patients were treated in a single fraction.^57^ To date, there are no comparative clinical data on the safety and efficacy of other doses or fractionation strategies. Target volume coverage requirements were described in eight series, which was a dose of 25 Gy covering 95% of the volume in 7/8 and 98% of the volume in 1/8, respectively.^20,30,31,36,58–62^

Only few data on the used dose constraints (target and OAR) or the measured dose on the OAR have been published. The maximum dose measured on the PTV was on average 29.2 Gy (median of 30.3 Gy; range 16.8–34.3 Gy), and the minimum dose was on average 16.7 Gy (median 19.4 Gy; range 5.8–24.4 Gy). Apart from the publications requiring 95% PTV coverage with at least 95% of the prescribed dose (PTV V95% = 95%), only two publications propose PTV constraints. Only nine publications provide data on the constraints used on OARs or on the doses actually measured.^20,26,58–60,63–67^ Reported data are heterogeneous. For example, for myocardium, 13 different OAR constraints have been proposed, without considering the valves or coronary arteries. A total of 61 constraints have been published for 20 different OARs, two of them for ICDs (see Supplementary material online, *Table S1*).

### 3.3. Target volume definition for stereotactic arrhythmia radioablation

Pre-treatment planning and treatment require close collaboration between specialists in cardiac electrophysiology, cardiac imaging, radiation oncology, and radiation physics. Centre-specific workflows have been used to transfer clinically collected electrophysiological data (the ‘VT substrate’) and/or pre-procedural imaging data, e.g. CT, cardiac magnetic resonance (CMR), and positron emission tomography (PET) to the planning CT with the aim to collaboratively delineate a suitable cardiac target volume. None of the existing target volume definitions in conventional radiation oncology [International Commission on Radiation Units (ICRU) 50,^68^ 62,^69^ 71^70^ 78,^71^ and 83 report^72^) apply well for cardiac purposes.

As several specialists with different backgrounds are involved in planning and implementing STAR, clear definitions and common nomenclature are desirable.

**Figure 1:**
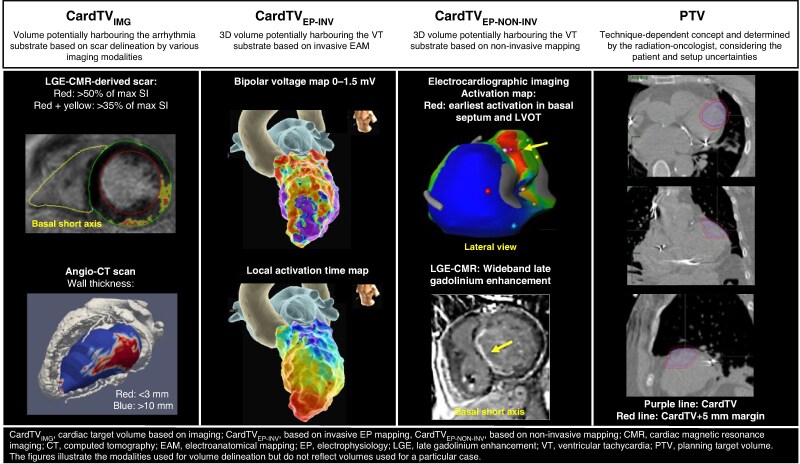
Suggested volume definitions for STAR based on imaging and/or electrophysiological evaluation.

For STAR, it is advised to provide the method(s) used to delineate the target volume and the use of the following uniform definitions (*Figure 1*).

Cardiac target volume (CardTV) is defined as the three-dimensional (3D) volume of the VA substrate as designated by the treatment team based on all available electrophysiologic and imaging data.

Cardiac target volume imaging (CardTV-IMG) is defined as the target volume potentially harbouring the arrhythmia substrate based on imaging. It is advised to specify the image modality used and the applied method and threshold to define scar or fibrosis (see Section 4.4.1).

Cardiac target volume electrophysiology (CardTV-EP) is defined as the volume of the VAs substrate delineated by invasive (CardTV-EP_inv_) or non-invasive (CardTV-EP_non-inv_) EAM of the endocardial and/or epicardial surface. It is desirable to provide detailed information on how the mapping data have been interpretated and have contributed to the CardTV-EP. For the CardTVs, it is advised to include safety margins that exclude healthy functioning myocardium.

## 4. Identification of the cardiac target volume

### 4.1. Relation between ventricular tachycardia and myocardial scar

Ventricular tachycardia associated with SHD is usually due to re-entry in regions of myocardial scar from prior infarction (ICM), inflammation, infiltrative disease, or prior surgery. In ICM, scars follow a coronary regional distribution pattern, with sub-endocardial predominance that may extend transmurally. In NICM, scar locations and architecture are more variable and can be endocardial, intramural, or sub-epicardial with focal or diffuse architecture.^73,74^ Areas with slow conduction within the scar are key for the initiation and maintenance of re-entrant VT and constitute targets for CA.^75^ The VT circuit may exist on the endocardium or epicardium, but commonly portions of the circuit or even the entire circuit are intramural such that the circuit cannot be completely defined from endocardial or epicardial mapping. In patients with scar-related VTs, programmed stimulation often induces more than one morphology of monomorphic VT. Multiple morphologies of VT may originate from different re-entry circuits or different exits from one culprit region. Of note, myocardial scar does not equal substrate for VT and accurate identification and delineation of ‘arrhythmogenic scar areas’ is desirable to allow precise treatment planning.

### 4.2. Clinical or presumed clinical ventricular tachycardia

Monomorphic VTs can be defined by their QRS morphology and rate. A clinical VT is one that has been documented to occur spontaneously.^76^ Ventricular tachycardia that is induced by programmed stimulation but has not been observed to occur spontaneously has been designated as non-clinical or non-documented VT. Persistent inducibility of the clinical VT after ablation has been associated with high VT recurrence rates.^77^ The presence of inducible non-clinical or undocumented VTs after RFCA is associated with increased VT recurrences suggesting that some non-documented VTs may subsequently occur spontaneously.^78–80^ However, one study found no increased risk of occurrence when non-documented inducible VTs were relatively fast [cycle length ≤ RV effective refractory period + 30 ms].^81^ For scar-related VT, the QRS morphology often reflects the location of the re-entry circuit exit along the scar border.^82,83^ The 12-lead ECG of spontaneous VT is often not available in patients with ICDs, but the VT cycle length and far-field and near-field electrograms from the ICD can be obtained and compared with those of VTs induced in the electrophysiology laboratory. In 21 patients with post-infarct VT, comparison of ICD electrograms with induced VTs allowed recognition of 98% of clinical VTs; the cycle length alone was not a reliable indicator of a clinical VT.^84^ Whether targeting non-documented inducible VT morphologies for STAR treatment has an impact on outcome is not known. Of note, ∼10% of patients with ICM or NICM do not have inducible VT despite spontaneous events,^85–87^ which limits non-invasive substrate identification based on body surface potential mapping.

### 4.3. Invasive mapping to identify scar and the ventricular tachycardia substrate

#### 4.3.1. Principles of electroanatomical mapping

Electroanatomical mapping is a critical component for modern-era arrhythmia (substrate) localization. The data acquired from these systems have been successfully incorporated into target planning for STAR.^4,5,22,23,26,29,57,60,88^ Invasive EAM systems use electromagnetic fields, and/or impedance measurements to localize catheters and render them in virtual space, such that electrogram voltage, wavefront activation/timing, and other electrophysiologic parameters can be displayed in 3D and used to inform STAR target area definition. Electroanatomical mapping quality depends on the technical features, point density, spatial resolution and stability, capabilities of the staff, and accuracy of near-field electrogram identification (*Table 7*).

**Table 7:** Electroanatomic mapping and ECGI systems, functionality, and special considerations

	Functionality	Features	Potential advantages	Data transfer	Potential limitations
**Non-invasive**					
Cardio-Insight Medtronic, Minneapolis, MN, USA	ECG vest integrated with CT to generate EAM	252 electrode vest with proprietary workstation to generate wavefronts and exit sites of arrhythmia	Non-invasive		Reduced ability to identify endocardial/septal targets Logistically complex workflow Limited evidence of its use with STAR
**Invasive**					
EnsiteNavx® EnsitePrecision® Abbott Inc., Abbott Park, IL, USA	Impedance based localization with combined magnetic adjunct on newer models (EnsitePrecision®)	Numerous automated post-processing features to identify substrate Proprietary multi-electrode catheter to refine local EGMs Automated morphology matching	Allows for contact or non-contact mapping Automated mapping post-processing options to identify arrhythmia exit sites and potential VA substrates	Data can be exported to a single .xml file containing triangulated mesh with associated interpolated electrophysiology properties. The data can be converted to a .vtk format.	Deterioration of maps with patient movement deep respiration No real-time ICE or fluoroscopic adjunct
CARTO® Biosense Webster Inc., Diamond Bar, CA, USA	Magnetic based localization with ultrasound adjunct	Several automated post-processing features to identify arrhythmia wavefronts Automated morphology matching	Stable, high-quality spatial resolution of anatomic structures ICE and fluoroscopic colocalization of anatomic structures Mapping post-processing options to identify arrythmia exit sites and potential VA substrates Used for STAR planning in most reports	.mesh files can be exported containing triangulated mesh of anatomical structures with interpolated EP data In addition, a point cloud with associated EP data and custom-made labels can be exported as text file. Exported data can be converted to formats readable by 3D image processing software's (e.g. .vtk)	Limited ability to localize several catheters at once Limited ability to integrate with non-proprietary catheters
Rythmia HDx™ Boston Scientific, Natick MA, USA	Combined impedance and magnetic localization	Proprietary mapping electrodes to refine EGM quality Automated EGM annotation Miniaturized electrodes	Rapid EGM acquisition Possible enhanced EGM signal to noise Increased point density	Limited export functionality. Generation of unstructured binary .xml format files, which can be converted to a readable .vtk file	Limited applicability of certain mapping catheters in epicardial space

This is a non-comprehensive table of commercial systems used in most electrophysiology laboratories.

CT, computed tomography; EAM, electroanatomical mapping; ECG, electrocardiogram; EGM electrogram; ICE, intra-cardiac ultrasound; MRI, magnetic resonance imaging; STAR, stereotactic arrhythmia radioablation; VA, ventricular arrhythmia.

#### 4.3.2. Voltage mapping

The first step in substrate mapping for scar-related VTs is to distinguish scarred tissue from healthy myocardium. Electrogram voltage recorded from a contact mapping catheter is linearly related to the amount of viable myocardium in the vicinity of the recording electrode.^74^ With the replacement of excitable myocardium by fibrosis, local EGM amplitudes decrease. Bipolar voltage is also influenced by electrode size, interelectrode distance, filtering, and wavefront direction.^89–91^ Accordingly, catheter and electrode-specific voltage thresholds are needed. Initial studies in young to middle-aged patients without SHD have shown that >95% of bipolar and unipolar electrograms recorded from standard tip electrodes exceeded 1.5 and 8.3 mV, respectively (see Supplementary material online, *Table S2*).^92^ A voltage threshold < 0.5 mV has been arbitrarily defined and commonly considered to represent dense scar, but this does not reliably indicate that myocytes and re-entry circuit channels are absent.^93,94^ Minimally filtered unipolar recordings have a greater field of view (FOV) compared with bipolar recordings and may allow recognition of replacement of viable myocardium by intramural or epicardial scar from endocardial recordings.^95–98^ Of note, in ICM and NICM, VT substrate can occur in regions with ‘normal’ endocardial voltage, defined by thresholds of 1.5 mV bipolar voltage (BV) or 8.3 mV unipolar voltage (UV), respectively.^87,99^ A head-to-head comparison with late gadolinium enhancement CMR (LGE-CMR)-derived scar localization in ischaemic heart disease has suggested that a BV threshold of >3.0 and >2.1 mV, respectively, may be more accurate to delineate LV areas without confluent fibrosis for not remodelled and adversely remodelled LV,^100^ but other cut-off values have been suggested (see Supplementary material online, *Table S2*). In NICM, fibrosis can be highly variable, confluent, patchy, or diffuse.^74,97^ Large areas of the LV are often affected in patients who have VT.^74^

**Figure 2:**
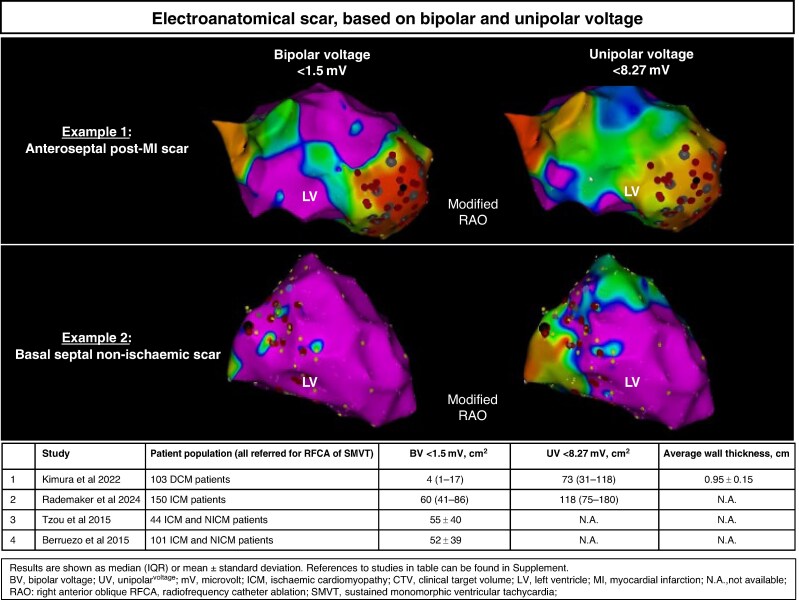
Electroanatomical scar size based on endocardial bipolar and unipolar voltage mapping for different aetiologies, according to suggested cut-off values for abnormal voltages.

**Figure 3:**
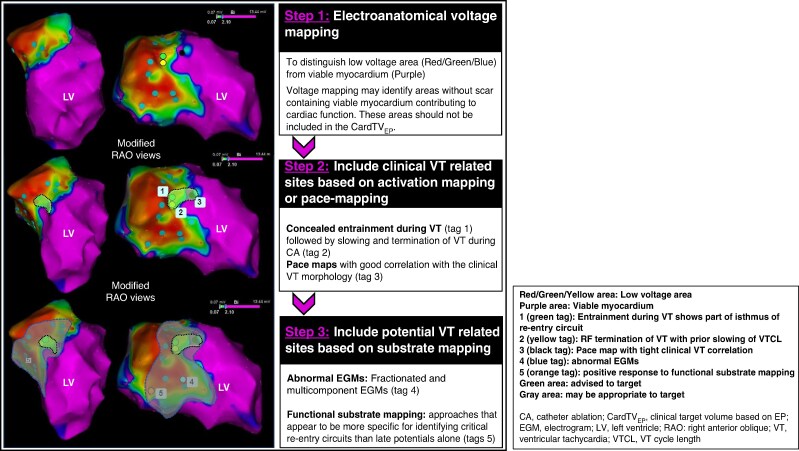
Cardiac target volume electrophysiology determination based on electroanatomical mapping.

Voltage maps derived from EAM data are often used to define the CardTV, but specific voltage threshold criteria for different diseases and mapping systems and electrodes to guide STAR have not been developed. The electroanatomical scar size varies depending on the use of UV or BV criteria (*Figure 2*). The accuracy of any single voltage threshold to detect fibrosis is limited, and to date, there is no voltage criterion for VT substrates. Stereotactic arrhythmia radioablation guided by voltage criteria alone has not been reported and is unlikely to reliably delineate relevant target areas. Delivering STAR only based on UV-derived scar delineation may result in large target volumes. However, voltage mapping may identify areas with significant viable myocardium contributing to cardiac function that should be excluded from the CardTV and may be considered the cornerstone for further mapping strategies (*Figure 3*).

#### 4.3.3. Pace mapping

Pace mapping (PM) in VT exit regions or at a focal arrhythmia source produces a QRS similar to that of the VT.^101,102^ For sub-endocardial ischaemic substrate, there is often a good correlation between PM at the re-entry circuit exit or within the central isthmus and the VT morphology.^101–106^ In one study of post-infarct VT patients, the estimated spatial resolution of PM for the VT exit region was 3.6 ± 4.5 cm.^84^ A stimulus-QRS latency exceeding 40 ms is consistent with slowed conduction through a channel within scar.^101–105^ Pace mapping might not reveal isthmuses bounded by functional conduction block, and the QRS morphology may fail to replicate the VT QRS at some re-entry circuit sites.^101,102,104^ For epicardial or intramural re-entrant scar-related substrate, PM can indicate the sub-endocardial exit site; however, this can be several centimetres distant from the critical isthmus.^105,106^ Maps displaying the correlation of paced QRS morphology with VT can reveal sub-endocardial areas of conduction block and re-entry isthmuses in some patients.^104^ The utility of PM in non-ischaemic dilated cardiomyopathy is less certain due to the variability in patterns of fibrosis. Pace mapping has been used to inform STAR target definition.

#### 4.3.4. Electrogram characteristics

Fractionated, multi-component electrograms, and late potentials during sinus or paced rhythm are evidence of long conduction times and discontinuous conduction through fibre bundles consistent with scar-related VT substrate. However, they are often present over a wide area of scar and have poor specificity for identifying specific re-entry circuits precisely^107^ and may fail to recognize intramural substrate. Successful abolition of late potentials and any local abnormal ventricular activity with CA has been associated with a lower risk of VT recurrence.^108,109^ High-density maps of sinus or paced activation have also been used to detect regions of slow activation, displayed as isochronal crowding that is associated with re-entry circuit isthmuses.^110,111^ Findings can vary with different activation sequences. To date, there is no firm evidence that high-density mapping provides better results for the identification of the arrhythmogenic substrate than point-by-point mapping with a solid tip catheter.

#### 4.3.5. Functional substrate mapping

Functional mapping approaches aim to identify areas where myocyte bundles are poorly coupled or dissociated as indicated by a change or emergence of split or late potentials or the propagation pattern in response to extra-stimuli, variation in pacing cycle length, or a different activation sequence.^87,108,110–117^ These methods appear to be more specific for identifying critical re-entry circuit sites than late potentials alone and can identify abnormal substrate in areas of relatively preserved bipolar voltage that occur in areas where a rim of functioning myocardium overlies fibrosis. The majority of patients studied had ICM in whom ablation targeting these potentials has been associated with favourable outcomes.^87,111,113,114,116,117^

#### 4.3.6. Mapping during ventricular tachycardia

Many STAR case series describe the use of invasive EAM including activation mapping^4,24,25,28–30,32,35^ and entrainment mapping^4,24^ usually combined with substrate mapping and/or imaging to plan the STAR target. Activation mapping combined with entrainment mapping followed by slowing and termination of VT during CA can be considered a gold standard to prove that a site is critical for the VT under investigation. However, identification of VT exit sites, outer loop sites, or sites where longer RF applications slow and terminate VT without eliminating inducibility may contribute to understanding the location of critical VT substrate that is not directly accessible to a mapping catheter. High-density EAM systems facilitate activation mapping of VT and localization of the critical isthmus.^106,118^ The majority of patients who are referred for STAR have failed RFCA and may be more likely to have an inaccessible substrate or are too frail to tolerate mapping during VT. Although the utility of mapping during VT to define STAR targets is not presently defined, previous activation and entrainment mapping data may facilitate the identification of scar areas harbouring the VT substrate.^25^

Target localization for the treatment of VAs by STAR has, in most reports, been based on a combination of anatomical and invasively collected electrophysiological information aimed at identifying the arrhythmogenic substrate.^4,23,24,26,27,29,32–36,66,119–122^ Only a few patients have undergone STAR guided only by the ECG of induced VT and imaging. However, invasive mapping data have been available in the majority of these patients, although in some of them, image integration was not used.^5,34,61^

Although the optimal use of different mapping approaches for guiding STAR has not been specified, it is advised to indicate sites based on activation mapping, PM, RF response, and functional information that are considered relevant by the EP on the EAM in patients who are considered candidates for STAR. It is also advised to incorporate invasive mapping data for the determination of CardTV-EP until data are available showing the reliability of non-invasive methods.

#### 4.3.7. Preparation for data transfer

**Figure 4:**
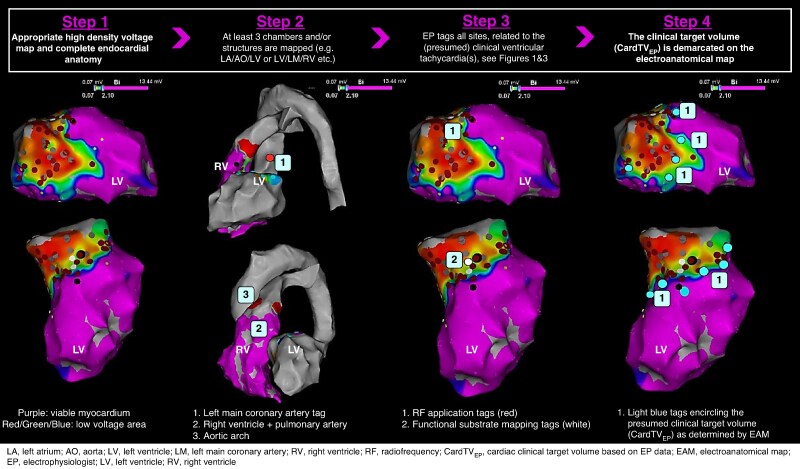
Suggested workflow for CardTV-EP delineation in preparation for STAR.

For accurate treatment of the target substrate, it is important to align the EAM with the CT scan. It is advised to obtain a detailed EAM, whenever possible with (i) appropriate and sufficient point density covering the (endocardial) surface of the chamber(s) of interest, (ii) anatomical marking of at least three chambers/distinct landmarks [e.g. LV/aorta/pulmonary artery (PA) or LV/left main (LM)/PA),^25^ and (iii) tagging all the sites that are related to the (presumed) clinical VT(s) or are considered as potential substrate based on EAM data as assessed by the treating electrophysiologist with patient-specific tags that allow for retrospective review of the target area (*Figure 4*).

### 4.4. Non-invasive methods to identify scar and the ventricular tachycardia substrate

#### 4.4.1. Imaging

##### 4.4.1.1. Imaging of scar

Cardiac magnetic resonance has become the gold standard for cardiac function assessment and non-invasive tissue characterization, in particular for the identification of areas of scar or fibrosis. However, one important drawback of CMR in this particular patient population is the high prevalence of ICDs or other indwelling devices that create significant imaging artefacts.

**Figure 5:**
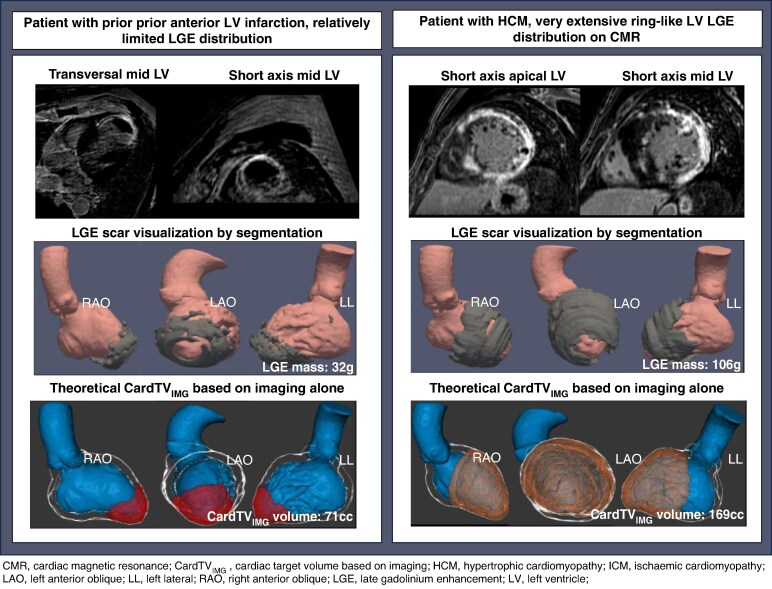
Theoretical CardTV_IMG_ if based on LGE-CMR for two different aetiologies.

**Figure 6:**
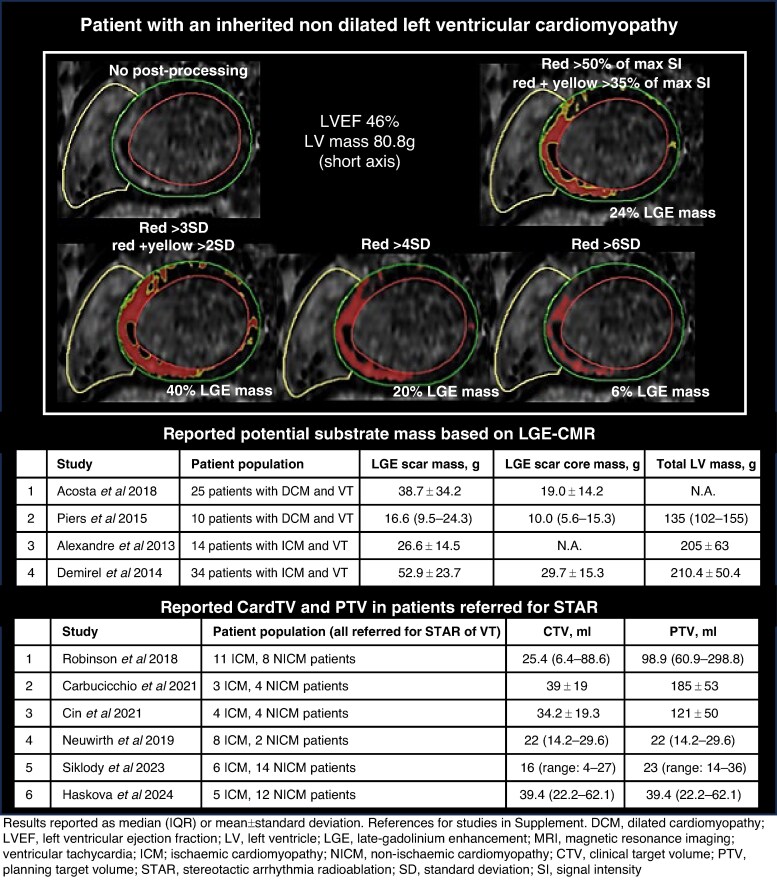
Variation in LGE based on threshold variation and reported CardTV-IMG based on LGE-MRI.

Late gadolinium enhancement is highly accurate and reproducible for visualizing macroscopic myocardial fibrosis (i.e. focal fibrosis/scar) (*Figure 5*),^123,124^ but, because of limited spatial resolution (1–2 mm range), characterizing tissue heterogeneity using LGE-CMR remains challenging.^125^ Late gadolinium enhancement does not reliably identify microscopic fibrosis (i.e. diffuse or patchy interstitial fibrosis), but this can be detected by T1-mapping CMR, albeit with low spatial resolution.^124^ Cardiac tissue characterization by CMR (both with T1-mapping and LGE-CMR) has been validated histologically for the presence of fibrosis/scar in ICM, but less robust for the different NICM.^126–130^ In addition, there is no general agreement on the method and threshold used to quantify non-ischaemic fibrosis (*Figures 5* and *6*).

During VT ablation procedures, electroanatomical voltage mapping (EAVM) is commonly used to detect scars.^94,96,131^ A fair correlation between EAVM and CMR-defined scar has been reported in several studies,^110,132–136^ but LGE-CMR is superior to EAVM in patients with intramural, small, or more heterogeneous scars.^114,133,137^ Patients undergoing STAR have generally failed prior CA guided by invasive mapping. In some cases, this may be due to intramural scar that escapes detection of mapping from the endocardium but might be appreciated by imaging.^138^

Cardiac CT (CCT) imaging is increasingly being implemented in clinical routine due to advances in technology offering high spatial and temporal resolution and diagnostic image quality and is ordinarily used for the non-invasive assessment of epicardial coronary arteries, to rule out LV thrombus and to assess epicardial fat. Although CCT is less able to characterize tissue compared with CMR, some techniques using late acquisition sequences, wall density, and thickness have been useful in guiding RFCA.^139,140^ Data from small observational studies have shown a moderate to good correlation between areas of wall thinning (<5 mm) on CCT and bipolar electrograms (BV < 1.5 mV) recorded on EAM.^139,141,142^ Of note, CCT is less affected by artefact from the ICD (although it may be affected by lead artefacts) and does not have safety concerns compared with CMR imaging in patients with older systems. Recent innovations have reduced radiation exposure.^143^ Drawbacks include the unfavourable signal-to-noise ratio conditioning suboptimal results particularly for chronic scars and the need for high doses of concentrated iodine-based contrast agents.

Advanced delayed enhancement CCT techniques with dedicated acquisition and post-processing algorithms are now under development and are expected to provide better tissue characterization and anatomical delineation of the scar in patients with ICM and NICM.^141,144^ Initial experience has reported excellent diagnostic accuracy for detecting myocardial fibrosis compared with EAM, suggesting that it could have a role for pre-procedural planning in the future.^144^

Nuclear imaging modalities, including both single-photon emission CT (SPECT) and PET, have shown utility in the delineation of areas potentially related to VA. A unique strength of these imaging modalities is the ability to assess a range of characteristics of the myocardium through the use of different tracers. The extent of perfusion defects and myocardial blood flow abnormalities defined by PET or SPECT has been associated with increased risk of VA.^142,145–149^ Several studies evaluating alterations in innervation, particularly in peri-infarct zones, have suggested that perfusion–innervation mismatch, particularly at the border of scar regions, may be associated with increased risk for VA.^147,150–153^ Inflammation, as assessed by fluorodeoxyglucose-PET (FDG-PET), has been associated with increased risk of VA particularly in cardiac sarcoidosis (CS).^154–158^ The incremental value of differing nuclear imaging modalities and their utility for guiding STAR is unclear, and further comparative investigation is needed.

##### 4.4.1.2. Imaging of ventricular tachycardia substrate

Magnetic resonance imaging (MRI)-detected scar is not synonymous with unexcitable tissue and cannot exclude the presence of surviving myocytes, within and at the BZs of dense scar, that are considered the anatomic substrate of VT in ICM.^68,153,154^ Scar detected by LGE-CMR matches with areas that contain critical sites for the maintenance of VT in studies with CMR co-registered with VT or substrate mapping.^159–163^ More precise characterization of the arrhythmogenic substrate, by using a signal intensity-based algorithm to identify potential conducting channels in scars, has shown promise in MRI-guided VT ablation procedures.^99,164,165^ Finally, pre-procedural substrate characterization with LGE-CMR to guide VT ablation has been associated with improved outcomes in some retrospective observational studies.^99,162,165–167^

It may be reasonable to deliver STAR to a limited scar area, in particular to avoid collateral damage, but this remains to be proven clinically. Large LGE volumes preclude imaging-based STAR (*Figure 5*, Case 4). A strategy of homogenization of low voltage areas of scar with RFCA has been superior to a very limited CA strategy in non-randomized studies.^168,169^ However, whether scar homogenization can be achieved by STAR is unknown. Therefore, the need for complementary information, such as the location of potential conducting channels,^170^ the geometry of scars,^132^ or CMR-based computational models of VT,^171,172^ remains to be assessed compared with LGE-CMR alone to guide VT ablation procedures, especially for STAR. In non-dilated LV cardiomyopathy and dilated cardiomyopathy (DCM), histological data on the substrate for VT are lacking. Late gadolinium enhancement cardiac magnetic resonance has limited ability to delineate some regions of fibrosis related to VT, and its value for guiding STAR is also unknown.^74^ It is not advised to deliver STAR solely based on LGE-CMR in patients with non-ischaemic fibrosis for all aetiologies considering the variation in amount and distribution of LGE and the uncertainty in correct delineation of non-ischaemic fibrosis related to VT (*Figures* 5 and  *6*). Although LGE-CMR has been validated to accurately delineate scar after MI, delivering STAR only based on LGE-CMR-derived scar delineation may result in undesirable large target volumes.

Contrast-enhanced multi-detector CCT has become an alternative imaging technique for delineating post-infarct scars just by the assessment of myocardial wall thinning. Although the majority of abnormal electrograms consistent with slow conduction have been observed to be located in areas of wall thinning,^97,139,140,142^ ordinary CCT imaging techniques alone are currently insufficient to identify scar segments that are required to sustain re-entrant VT. Nevertheless, the majority of ablation target sites for post-infarct VT have been identified within so-called CCT channels, defined as abnormal corridors bordered by thinner areas.^97,142^ This method does not seem to be useful for identifying substrate in NICM.^140^

Nuclear imaging modalities including PET and SPECT have been shown to correlate to invasive EAM data and localization of areas harbouring VT circuits. In correlation to surgical mapping data, VT circuits were localized predominantly to the peripheral of areas of perfusion deficits.^173^ Subsequent investigation has shown good correlation of SPECT-defined areas of perfusion deficits with endocardial voltage abnormalities.^174^ Studies evaluating the correlation of PET imaging, in particular perfusion imaging, to findings during invasive mapping showed a generally good correlation with low voltage regions and potential utility in identifying areas of non-transmural scar and metabolically active channels in scar areas that may harbour VT circuits.^175–180^ Further studies have suggested that there may be additive value in PET imaging evaluating innervation defects for localization of VT substrate.^181,182^ To date, there are no data on mapping and ablation of VT guided by nuclear image modalities.

#### 4.4.2. Non-invasive definition and delineation of the ventricular tachycardia substrate by (body surface) electrocardiography

One potential advantage of the use of radiotherapy for the treatment of VT is the avoidance of cardiac instrumentation, with its attendant procedural risks, provided that the CardTV-EP can also be accurately delineated non-invasively. The standard ECG provides useful information to localize the potential VT exit area. It can be enhanced with the incorporation of individualized patient anatomic imaging and with the addition of multiple thoracic electrodes (body surface potential mapping) to calculate estimated epicardial cardiac activation origins [ECG imaging (ECGI)].^183^ Several studies have aimed to validate ECGI against contact mapping data (see Supplementary material online, *Table S3*). In general, these methods have limited efficacy for the localization of myocardial scar and have greater efficacy in localization of the epicardial origin of cardiac activation when it is extrinsic to the cardiac conduction system.^184^ There are three commercially available non-invasive ECG-based mapping systems that have been used to non-invasively guide STAR: a forward-solution 12-lead ECG computer simulation-based mapping (vMap, Vektor Medical Inc.), simulated activation-based 12-lead ECG mapping (VIVO, Catheter Precision Inc.), and an inverse solution 252-electrode body vest and ECGI system (CardioInsight, Medtronic) (*Table 7*). The vMap system has been used in a few patients to guide STAR of VT utilizing the forward-solution and a biventricular library of computer simulations.^29^ This method localizes the earliest site of activation during VT or PVCs based solely on a standard 12-lead ECG.^185^ Median spatial accuracy for VT was reported to be 14 mm with excellent regional localization in patients, 23% of whom had SHD, who were undergoing RFCA. The VIVO system uses the 12-lead ECG integrated with personalized lead position (verified with a 3D camera) and personal cardiothoracic anatomy (derived from CT or MRI) demonstrated very good regionalization of cardiac arrhythmias^186^ and has also been used to guide STAR.^30^

Electrocardiogram imaging, one commercially available system (CardioInsight, Medtronic), applies inverse solution mathematics to electrograms recorded from an array of 252 body surface electrodes to reconstruct epicardial surface unipolar electrograms that are projected onto an epicardial geometry. Challenges include correction for cardiorespiratory motion artefact, and the heterogeneous tissue impedance characteristics between the skin and heart. The method largely reflects the epicardial activation sequence and the epicardial exit of the re-entry circuit, which can be more than 3 cm distant from the critical re-entry circuit isthmus.^187^ This is also a significant limitation for septal VT substrates. Initial comparisons with epicardial recordings indicated accuracy within 1 cm^188–190^ and compared well with sites of successful RFCA.^191^ In a human validation study in patients undergoing epicardial VT mapping and ablation procedures, the mean localization accuracy was 13 mm and was reduced by the presence of nearby scar. The activation maps were qualitatively similar but showed variation including abrupt changes in activation times giving the appearance of lines of block and rapid activation over large areas of myocardium.^192^ These latter two features have also been observed in other studies.^188,189,193^ Large epicardial areas activating simultaneously would impact the accuracy of using the system to locate exit sites for VT (for example in the case of an endocardial site of origin). Artefactual lines of block on the other hand could falsely identify areas as zones of slow conduction, hindering the capability to risk stratify patients for arrhythmia or locate VT circuits. During intra-procedural use of ECGI to localize VT sites of origin during CA in structurally abnormal hearts, the median accuracy was 22.6 mm (IQR 13.9–36.2), and the predicted anatomic segment for VT was correct for 83.3% of VTs studied.^194^

Electrocardiogram imaging has been utilized in conjunction with non-invasive programmed stimulation to identify VT exit sites for the purpose of guiding STAR and was performed on all patients in the first series of patients reported undergoing this therapy. Although this study did not include a mechanism for validation of regional localization, the good results achieved suggest that it was an effective strategy.^5,23^

The level of accuracy achieved by ECGI in localization of VT exit from myocardial scar appears to be adequate to guide a regionally directed therapy, such as STAR. It is not yet clear whether this provides a clinically significant advantage over the standard ECG, with or without individual anatomic personalization, for guiding STAR.

## 5. Patient selection for stereotactic arrhythmia radioablation

Both AAD therapy and RFCA are standard accepted standard therapies for VAs. Radiofrequency catheter ablation has been proven effective for the reduction of ICD shocks and VT burden in large cohorts of patients with various SHDs.^195^ In contrast, data on the efficacy and long-term safety of STAR for the treatment of VT are limited to small, mainly retrospective case series (*Table 4*). Therefore, it is advised to consider STAR either in the context of an approved investigational trial or as a bailout strategy with appropriate institutional approvals for patients with VT refractory to or with contraindications for (dose-escalated) AAD and RFCA performed in an expert centre. The latter is an important requirement since only such centres are able to offer the spectrum of alternative strategies. The potential role and benefit of STAR for different aetiologies of heart disease is unclear. In some patients, limitations of RFCA may justify consideration for STAR despite the limited long-term outcome data on efficiency and safety.^85,196–198^

### 5.1. Ischaemic cardiomyopathy

The arrhythmogenic scar in post-MI patients can be effectively treated by conventional RFCA in the majority of cases. Favourable results are related to its anatomical characteristics, its sub-endocardial localization in areas with wall thinning, and the limited involvement of intramural layers.^76,77^ A trans-septal access, substrate-based ablation without the need of repeated VT induction, short procedure times, and low reported complication rates allow for safe ablation in the majority of post-MI patients and is a recommended treatment option in the 2022 European Society of Cardiology (ESC) guidelines for the management of patients with VAs and the prevention of sudden cardiac death.^77^ Procedural failure can be related to anatomic barriers prohibiting adequate lesion formation or occasionally due to a non-endocardial VT origin.

Stereotactic arrhythmia radioablation has been considered as a bailout procedure (*Table 4*) for (i) patients with LV endocardial mobile or large laminar thrombus despite anticoagulation, which is associated with embolism risk or may prevent endocardial catheter access to the arrhythmia substrate; (ii) patients with inaccessible arrhythmia substrate due to intramural or epicardial substrate when percutaneous epicardial access is limited (e.g. after coronary artery bypass grafting) or mechanical valves; and (iii) patients deemed to be frail with high-procedural risk for RFCA or surgical ablation. This group has been less well defined. Stereotactic arrhythmia radioablation must be carefully evaluated balancing pros and cons, also taking into account other potential treatment modalities and strategies to increase lesion size (e.g. percutaneous or surgical epicardial access, trans-coronary, and venous alcohol ablation).

### 5.2. Non-ischaemic heart disease

Non-ischaemic aetiologies encompass a variety of morphological and functional phenotypes. Non-ischaemic cardiomyopathy often refers to DCM, which itself includes distinct underlying aetiologies such as CS, myocarditis, or genetic diseases. Prognosis varies across aetiologies and severity of heart disease. Approximately half of the patients with recurrent VT benefit from currently available catheter mapping and ablation techniques.^85,86,199–201^ Aetiology of NICM is a significant predictor of CA outcomes, with arrhythmogenic RV cardiomyopathy (ARVC) and myocarditis having similar but superior outcomes to hypertrophic cardiomyopathy, valvular cardiomyopathy, sarcoidosis, and inherited (genetic) cardiomyopathy.^201–203^ Stereotactic arrhythmia radioablation has been used as a salvage treatment modality for refractory VT in NICM of different aetiologies (*Table 4*). Further work is needed to determine the efficacy and safety of STAR in these different disease entities.

#### 5.2.1. Dilated cardiomyopathy

Disease progression and the complex underlying substrates with patchy or diffuse fibrosis in locations that are often not reachable by RFCA contribute to procedural failure and recurrences during long-term follow-up. Of note, DCM patients with early VT recurrence after RFCA and LVEFs < 32% had a 1-year rate of mortality or heart transplantation of 55%, mainly due to progressive heart failure in a large multi-centre study, which is important to keep in mind if STAR is considered in these patients.^86^ Although significant reductions in ICD therapies were reported in most series, long-term VT suppression with STAR in DCM remains difficult with most patients experiencing recurrences. Stereotactic arrhythmia radioablation may be appropriate for refractory VT in patients with DCM if the arrhythmogenic substrate can be localized. Options for the management of advanced and progressive heart failure need to be discussed. Whether potential consequences of irradiation (e.g. post-pericarditis adhesions) will impact LV assist device or cardiac transplantation after STAR is currently not known.

#### 5.2.2. Sarcoidosis

There are limited data on the use of STAR in patients with CS. These patients are a small fraction of those reported (see Supplementary material online, *Table S4*).^120^ Stereotactic arrhythmia radioablation appears to be able to decrease VT burden and ICD therapies, but more data are needed. The arrhythmia substrate location in CS is variable and can be patchy.^203,204^ Prior studies of patients undergoing RFCA have described a median of three induced VT morphologies often from anatomically disparate locations.^205,206^ In one retrospective evaluation of patients with CS undergoing VT ablation, Muser *et al*.^206^ found that abnormal electrograms on invasive mapping were more likely to be found in LGE-positive/FDG-negative segments and in areas of greater scar transmurality. Radiotherapy has been described to modulate immune signaling.^207^ However, it is unclear whether (additional low dose) radiation is beneficial in patients with active sarcoidosis. Patient-specific approaches utilizing multi-modality evaluation of VT substrate will be required with larger studies needed to guide planning optimization.

#### 5.2.3. Arrhythmogenic right ventricular cardiomyopathy

Among NICM, patients with VT due to ARVC have the most favourable outcome after CA.^201,208^ Ventricular tachycardia substrates are amendable to endo-/epicardial mapping and ablation.^209–212^ Procedural failure is mostly related to inaccessible epicardial substrates, i.e. due to epicardial fat or other anatomical constraints or progression of the underlying disease.^211^ Stereotactic arrhythmia radioablation has been reported in two patients^28,88^ with (presumed) epicardial/transmural substrate in the RV-free wall who had ≥3 failed endocardial RFCA procedures for incessant VT. Outcomes were favourable, establishing feasibility; however, further study and long-term safety data for the on average younger patients are needed.

#### 5.2.4. Hypertrophic cardiomyopathy

Sustained monomorphic VT in hypertrophic cardiomyopathy (HCM) can be related to an apical aneurysm that is often amenable to RFCA or arise from deep intra-septal and/or basal LV, which in combination with the given thickness of the LV and overlying fat in this region frequently hampers endo- or epicardial access.^201,213–216^ To address these challenging substrates, alternative treatment strategies may be necessary. Feasibility of STAR has been shown with favourable outcomes in a small number of patients including some with substrates at the LV apex.^25,28,36,217,218^

## 6. Target volume transfer

### 6.1. Image integration, data export, and inverse registration

A crucial step in STAR is the transfer of the CardTV identified by another modality (most commonly by invasive EAM) to a pre-procedural planning CT. In early reports, the CardTV has been manually delineated on the planning CT by side-by-side comparison with EAM data (i.e. ‘eyeballing’), using the tip of an indwelling ICD electrode for rough orientation with its immanent limitations. Treatment failure has been reported in cases due to missed VT substrate using this method.^42^

More recently, CardTV has been manually transferred to the American Heart Association 17 segment model to guide STAR.^37^ Detailed methodology was not provided, and it is unclear how functional myocardium was excluded. In general, it is not advised to deliver STAR to an entire LV segment of the standard 17-segment LV model, if the CardTV-EP determined by mapping involves only part of that segment; irradiation of viable myocardium should be minimized. Inaccuracy of CardTV-EP transfer to planning CTs is expected to increase the risk of STAR due to larger PTVs to compensate for the inaccuracy.^55,63^ A potential solution is to transfer the identified area of interest from the 3D EAM (CardTV-EP_inv_) to a planning CT using direct image integration. Co-registration of CCT or CMR with 3D EAM to guide VT ablation is a well-established and routinely used method and has been performed with high registration accuracy using landmarks and surface registration algorithms.^219^ The export of registered datasets (inverse registration) permits superimposition of the co-registered 3D mapping data directly on the image modality.^137^

Various additional methods and software tools have been proposed for image registration, also dependent on the 3D mapping system used. It is important to emphasize that no currently available software packages are approved specifically for the planning of STAR.

#### 6.1.1. Electroanatomical mapping systems used for image integration

Currently, there are three available invasive EAM systems that can be used to identify and transfer CardTV-EP_inv_ to the planning CT (*Table 7*). CARTO 3 (Biosense Webster) has been most widely used with published data on the registration accuracy of imaging and EAM data, also in the context of STAR.^4,25,220^ It allows the export of smooth anatomical models to .mesh files, which contain triangulated mesh coordinates of the anatomical structures with interpolated electrophysiology data such as voltage or local activation time for each vertex of the mesh. An additional point cloud of the original mapping points with associated electrophysiology properties and custom labels are exported as a text file. This file can be particularly useful for projecting individual points of interest (e.g. EAM-defined border of CardTV_EP_, ostium of the left main coronary artery, and conduction system location) onto the CT. Exported CARTO files are converted to a format readable by a 3D image processing software (most commonly to a .vtk file or to fiducial points for the 3D Slicer software). The conversion can be achieved by custom scripts,^25,221,222^ some of them implemented as a module for 3D Slicer software (www.slicer.org) or within standalone applications.^223^ It should be recognized that different specific versions of the CARTO 3 system vary in the scope of the exported data.

EnSite Velocity/Precision (Abbott, MN, USA) is an EAM system that exports data to a single .xml file containing triangulated mesh with associated interpolated electrophysiology properties. The data can be converted to a .vtk format with a free software tool.^222^ Experience with the use of EnSite for STAR is limited to a few published cases.^223^ Rythmia HDX (Boston Scientific) is an EAM system that currently does not have export functionality. It has been used only in one published case of STAR.^224^ The system can generate unstructured binary .xml format files, which can be converted to a readable .vtk file.

#### 6.1.2. Software for image registration

Most reports on image integration between EAM and planning CT for STAR used 3D Slicer, which is a freely available open-source software program for 3D image processing. It contains useful tools and algorithms and supports Python scripting and creating of custom modules. The software does not have FDA or CE Mark certification, and is currently used for research projects on STAR. ADAS 3D (Galgo Medical Barcelona, Spain) is a stand-alone commercially available software for the integration of MRI and CT images with all three EAM mapping systems. It has been used for image-guided VT ablation.^225^ Data related to STAR are limited to a few published cases.^221,223^ It has no FDA clearance or CE Mark Certification for planning of STAR. MUSIC (Multimodality Platform for Specific Imaging in Cardiology) is a software platform composed of a desktop application, a web portal, and an academic consortium. It enables the import of files from all three EAM mapping systems and can perform various registration tasks. Currently, it is available to consortium members.

Recently, a software tool has been proposed that allows the projection of two-dimensional (2D) screenshots from EAM images on 3D CT images. The method also involves projecting a 17-segment heart model on the EAM map to indicate the target for STAR. This approach for STAR has been reported for five cases.^224^ In this series, the CardTV-EP_inv_ was drawn manually on the planning CT, and image integration was used merely to verify the results, thereby overcoming concerns with non-certified software.

Registration of the EAM-derived and CT-derived anatomical structures for STAR has been most commonly performed manually, using various anatomical landmarks (e.g. the ostium of the left main coronary artery) combined with overlay of different anatomical structures (LV, RV, left atrium, and aortic arch).^25^ In a recent experimental study, this approach achieved good registration accuracy (median distance between modelled and registered gross target volume of <3 mm) and high inter-observer reproducibility. The best results were achieved (median distance error of 2.6 mm) by using at least three chambers for the registration in combination with the left main coronary artery. Of note, findings from studies on image-guided VT ablation showed that using fully automated algorithms without additional landmarks can lead to unacceptable registration (rotation) errors.^226^ For radiation planning, it should be kept in mind that larger registration errors seem to occur (due to shifts), when the target for STAR is located on the lateral wall or interventricular septum.

#### 6.1.3. Example of a workflow of image integration

The general workflow of image integration for STAR can be subdivided into four steps: (i) EAM-derived 3D models of anatomical structures are exported from an EAM system to a format readable by image processing software; (ii) using dedicated software, a 3D model of the heart is segmented from a contrast-enhanced CT and registered with the EAM-derived anatomical structures; (iii) CardTV-EP_inv_ is drawn manually on 2D CT images to obtain a transmural 3D structure. This process can be guided by projecting EAM-derived myocardial contours and the mapping points of interest or other substrate characteristics directly on the CT images. (iv) The CT image stack with delineated CardTV-EP_inv_ is exported as a DICOM file (DICOM-RT or plain DICOM with enhanced voxels) for further registration with a (non-contrast) planning CT in the SBRT workstation.^25,221,222,227,228^

### 6.2. From the angio-computed tomography to the planning computed tomography

A comprehensive workflow review including planning imaging and target definition has been published in 2021.^229^ The process input is an angio-CT (ECG-gated optional) and the CardTV-EP_inv_, while the process output is the planning CT with PTV specific to treatment modality and motion management approach. All types of planning CT must meet the general criteria such as sufficient coverage of organs whose radiation toxicity is based on dose–volume histogram-based parameters and sufficient coverage of all possible beam entries including non-coplanar geometries. High spatial resolution in transversal plane is desired—ideally <1 mm. If this is impossible for a large FOV to include whole patient body and immobilization devices, then retrospective reconstruction from the same projection data with small FOV around the target area is an option for more precise contouring on a secondary image without the need of registration. Slice spacing should be <2 mm, especially for imaging of the spine or fiducials and for better quality of digitally reconstructed radiography, if relevant for IGRT. On the other hand, with decreasing slice spacing, there is a decrease of contrast-to-noise ratio. Based on STAR modality and motion management approach, various types of planning CTs are relevant:

- Expiration breath-hold CT for treatments with CyberKnife tracking of ICD or pacemaker lead tip as a target surrogate or for same-phase-gated treatment on a C-arm linear accelerator.
- (Deep) inspiration breath-hold CT for same-phase-gated treatment on a C-arm linear accelerator.
- Average intensity projection CT or mid-ventilation CT derived from four-dimensional CT (4DCT) series or standard free-breathing CT for combined cardio and respiratory internal target volume (ITV)-based treatments on a C-arm linear accelerator.

In case of the use of 4DCT and its derivatives, depending on technology, attention to image quality should be given for the possibility of relatively increased noise and also residual motion artefacts. In case of the use of a single-phase CT at extreme respiratory phase (expiration and inspiration) and treatment at free-breathing, its representativeness during whole cycle must be assessed—especially potential deformations and effect on optimized and evaluated dose distribution. Acquisition of 4DCT or single-phase (breath-hold) CT assumes relevant respiratory phase monitoring technology based on either external monitors or internal image-based data processing.

The application of metal artefact reduction solutions should be considered for the acquisition of planning CTs in areas with metallic components. If applied, careful assessment of the potential impact on Houndsfield Unit-relative electron density and dose calculation should be carried out.^230^

As described earlier, the CT image stack with delineated CTV_EP_ from the 3D angio-CT is exported. This information needs to be further transferred and image registration is required. The accuracy of this process is affected by the differences between secondary and primary CT caused mainly by respective instant phases of both respiratory and cardiac motions. One could consider that cardiac motion is covered in non-ECG-gated ‘slow’ CT regardless of its type; however, using image registration with two ECG-gated CT scans in both systole and diastole is preferable as no cardiac tracking solution is currently available. The whole heart must be registered using rigid or deformable image registration (DIR) tools. Application of DIR requires careful quality assurance.^231^

When the planning CT is equivalent to the input angio-CT in terms of respiratory, cardiac, or both motions, registration uncertainty determining the accuracy of target volume transfer onto planning CT should be reduced or even eliminated.

When the CardTV-EP is transferred on the primary planning CT, following the target volumes concept of ICRU 62 (ICRU published in 1999 Report 62 suggesting various target as well as organ at risk volumes that are important in prescribing and reporting photon beam therapy), the ITV may be constructed based on predicted (residual) motion derived from complementary pre-treatment imaging (e.g. 4DCT) and finally the PTV, by adding the remaining uncertainty margin based on particular treatment modality, technique, and IGRT. Once the PTV is defined, the process continues with radiation treatment planning, i.e. optimization of dose distribution to meet target dose prescription and to minimize radiation toxicity to critical organs.

## 7. Future directions

Stereotactic arrhythmia radioablation is an emerging technique that may benefit from rigorous scientific study and iterative refinement. Its value needs to be viewed in the context of evolving developments in invasive electrophysiology, such as pulse field ablation or ultra-low cryoablation.^232^ High-quality observational and comparative studies should include comprehensive descriptions of patients’ clinical characteristics, details of concomitant anti-arrhythmic pharmacotherapies, and all procedural interventions. Complete follow-up and reporting of arrhythmia recurrences, survival and cardiovascular outcomes, careful follow-up of cardiac and valvular function, and complete reporting of complications of radiotherapy, ideally including longer-term follow-up and using common reporting terminology, are of utmost importance. Initiatives like the STOPSTORM consortium funded by the European Union's Horizon 2020 research and innovation programme (No 945119) will further contribute to the standardization of reporting and follow-up.

There remain several important questions that are yet unanswered. These may be addressed by the following:

- Studies of the mechanism of action by which radiotherapy influences cardiac rhythm. What are its effects on cardiac and myocardial cellular electrophysiology, ion channel expression and function? How does it affect cardiac microvasculature, inflammation and cardio neural systems?
- Investigation of optimal dose and fractionation regimen to achieve maximal effectiveness and safety. Can it be safely re-administered for recurrences or new VAs? What are the optimal dose, exact response, and time to effect in diseased human myocardium?
- Observational studies to understand the durability of effects, long-term effectiveness, and long-term safety.
- Careful assessment of both acute and longer-term toxicity and effects on coronary vasculature, myocardium, and valves.
- Rigorous study of optimal methods for delineating and targeting arrhythmogenic substrate to achieve effects with minimal risk. Should all scar regions be targeted? Should STAR be restricted to identify VT exit sites? Should all induced VTs regardless of cycle lengths and prior documentation be targeted? What are optimal methods for integrating cardiac imaging and electrophysiologic/functional studies to identify target sites? Is ECGI alone sufficiently reliable for defining the CardTV-EP?
- Randomized comparisons to other effective therapies, separately for different aetiologies including comparison of STAR to repeat CA for patients who have had prior CA and comparison of STAR to CA for patients who have recurrent arrhythmias despite AAD therapy who have characteristics suggesting high CA procedural risk. If it compares well in these patient groups, comparison with first-line VT suppressive therapy could be warranted.

Stereotactic arrhythmia radioablation holds significant potential as a non-invasive approach to treating malignant VAs. Ideally, its initial use will be when proven therapies are ineffective or unavailable, and expansion of its role in patient care will follow the development of high-quality, reliable clinical evidence.

## Supplementary Material

euae214_Supplementary_Data
