# Supplementary material for: Patient selection, ventricular tachycardia substrate delineation, and data transfer for stereotactic arrhythmia radioablation: a clinical consensus statement of the European Heart Rhythm Association of the European Society of Cardiology and the Heart Rhythm Society
Source: Europace. 2024 Aug 23;27(4):euae214. doi: 10.1093/europace/euae214 (PMC12041921; doi:10.1093/europace/euae214)
Supplement: euae214_Supplementary_Data [file euae214_supplementary_data.docx]

**Supplemental material**

Supplemental material

**Next page: Supplemental table S1: Measured doses or dose constraints proposed in the various publications on cardiac radioablation involving patients actually treated.**

| **Publication** | **Year** | **Heart V20** | **Heart Dmean** | **Heart V16** | **Heart Dmax** | **Heart D2%** | **Heart D50%** | **Heart-PTV D50%** | **Heart-PTV Dmax** | **Heart-PTV median dose (all patients)** | **Whole heart minus PTV D50%** | **Left atrium Dmax** | **Left atrium median dose (all patients)** | **Right atrium median dose (all patients)** | **Aortic valve Dmax** | **Aortic valve median dose (all patients)** |  |
| --- | --- | --- | --- | --- | --- | --- | --- | --- | --- | --- | --- | --- | --- | --- | --- | --- | --- |
| Chiu | 2021 | < 15% | < 2 Gy | < 15 Gy |  |  |  |  |  |  |  |  |  |  |  |  |  |
| Blanck | 2020 |  |  |  |  |  |  |  |  |  | ≤ 5 Gy | 4.4 Gy |  |  |  |  |  |
| Levis | 2022 |  | 3.15 Gy |  | 29.21 Gy | 24 Gy | 1.35 Gy |  |  |  |  | 16.78 Gy |  |  | 0.83 Gy |  |  |
| Krug | 2020 |  |  |  |  | 27.7 Gy | 5.2 Gy |  |  |  |  |  |  |  |  |  |  |
| Narducci | 2020 |  |  |  | 34.30 Gy |  | 11.95 Gy |  |  |  |  |  |  |  |  |  |  |
| Kluge | 2022 |  |  |  |  |  |  | ≤ 5 Gy |  |  |  | 4.4 Gy |  |  |  |  |  |
| Knutson | 2019 |  |  |  |  |  |  |  |  | 5.7 Gy |  |  | 3.6 Gy | 2.9 Gy |  | 3.5 Gy |  |
| Qian | 2022 |  |  |  |  |  |  |  | 29.9 Gy (29.8-30.6) |  |  |  |  |  |  |  |  |
|  |  | **Pulmonic valve Dmax** | **Pulmonic valve median dose (all patients)** | **Mitral valve Dmax** | **Tricuspid valve Dmax** | **Aorta Dmax** | **Aorta median dose (all patients)** | **Left coronary arteries Dmax** | **LMT Dmax** | **LAD Dmax** | **LAD median dose (all patients)** | **CFLX Dmax** | **CFLX median dose (all patients)** | **RCA Dmax** | **RCA median dose (all patients)** | **Pulmonary artery median dose (all patients)** |  |
| Blanck | 2020 |  |  |  |  | 20 Gy (constraint) |  | 14 Gy (constraint) |  |  |  |  |  |  |  |  |  |
| Levis | 2022 | 0.39 Gy |  | 27.69 Gy | 4.32 Gy | 2.96 Gy |  |  | 0.47 Gy | 9.31 Gy |  | 32.50 Gy |  | 1.28 Gy |  |  |  |
| Krug | 2020 |  |  |  |  |  |  |  |  | 12.8 Gy |  |  |  |  |  |  |  |
| Narducci | 2020 |  |  |  |  | 5.05 Gy |  |  |  |  |  |  |  |  |  |  |  |
| Kluge | 2022 |  |  |  |  | 20 Gy (constraint) |  | 14 Gy (constraint) |  |  |  |  |  |  |  |  |  |
| Knutson | 2019 |  | 1.8 Gy |  |  |  | 1.6 Gy |  |  |  | 10.1 Gy |  | 9.2 Gy |  | 3.2 Gy | 0.66 Gy |  |
|  |  | **Superior vena cava D50%** | **Superior vena cava median dose (all patients)** | **Esophagus Dmax** | **Esophagus Dmean** | **Esophagus V9** | **Esophagus D5 ml** | **Trachea Dmax** | **Trachea Dmean** | **Trachea V10** | **Bronchial tree Dmax** | **Bronchial tree V10** | **Spinal cord Dmax** | **Spinal cord Dmean** | **Spinal canal Dmax** | **Spinal canal Dmean** |  |
| Blanck | 2020 | ≤ 0.6 Gy (constraint) |  | 14.5 |  | ≤ 1 cc |  | 15 Gy |  | ≤ 1 cc | 15 Gy | ≤ 1 cc |  |  | 7 Gy |  |  |
| Levis | 2022 | 0.23 Gy |  | 5.86 Gy | 1.49 Gy |  |  | 0.37 Gy | 0.08 Gy |  |  |  | 1.11 Gy | 0.25 Gy |  |  |  |
| Haskova | 2022 |  |  | 13.9 Gy |  |  | 9.23 Gy |  |  |  |  |  |  |  |  |  |  |
| Narducci | 2020 |  |  | 5.01 Gy | 1.52 Gy |  |  |  |  |  | 0.77 Gy |  |  |  | 2.59 Gy | 0.67 Gy |  |
| Kluge | 2022 | ≤ 0.6 Gy (constraint) |  |  |  |  |  |  |  |  |  |  |  |  |  |  |  |
| Knutson | 2019 |  | 0.9 Gy | 11.9 Gy (constraint) |  |  |  |  |  |  |  |  | 10 Gy (constraint) |  |  |  |  |
| Qian | 2022 |  |  |  |  |  |  |  |  |  | 4.8 Gy (1.8-5.4) |  | 2.7 Gy (21.0-4.5) |  |  |  |  |
|  |  | **Spinal canal V6** | **Skin Dmax** | **Skin V10** | **Whole lungs V7 Gy** | **Whole lungs Dmax** | **Whole lungs Dmean** | **Whole lungs D50%** | **Whole lungs D5%** | **Whole lungs D2%** | **Lung-ITV V12** | **Stomach Dmax** | **Stomach PRV (Dmax)** | **Bowel Dmax** | **Liver V11** | **ICD (major electronics) Dmax** | **ICD Dmean** |
| Blanck | 2020 | ≤ 0.1 cc | 14.4 Gy | ≤ 10 cc | ≤ 1500 cc (constraint) |  |  | ≤ 3.5 Gy | ≤ 20 Gy |  |  |  |  |  |  | ≤ 0.5 Gy |  |
| Levis | 2022 |  |  |  |  | 19.66 Gy | 1.48 Gy | 0.16 Gy |  | 16.01 Gy |  |  |  |  |  | 0.04 Gy | 0.02 Gy |
| Cha | 2021 |  |  |  |  |  |  |  |  |  |  | 9.8 Gy | 14.6 Gy |  |  |  |  |
| Krug | 2020 |  |  |  |  |  |  |  |  |  |  | 13.8 Gy |  |  |  | 0.2 Gy |  |
| Narducci | 2020 |  |  |  |  | 28.96 Gy | 2.83 Gy |  |  |  |  |  |  |  |  |  |  |
| Knutson | 2019 |  |  |  | ≤ 1500 cc (constraint) |  |  |  |  |  |  | 17.4 Gy (constraint) |  |  | ≤ 700 cc (constraint) | 0.131 Gy (0.031-0.603) |  |
| Qian | 2022 |  |  |  |  |  |  |  |  |  | 4.4% (3.2-5.2) | 15.9 Gy (15.3-16.1) |  | 4.6 Gy (3.0-10.7) |  |  |  |

**Supplemental data on treatment and volume reporting Chapter 3**

In 10 series (1-10) patients were treated with CyberKnife. Volumetric modulated arc therapy (VMAT) was applied in 10 series.(11-22), In one series MRI-linac was used(23), and in 11 only generic data such as the manufacturer and model of the linear accelerator were provided, or simply CBCT based linac described (24-35). Apart from the use of real-time tracking (using the ICD as fiducial), among the patients treated with CyberKnife, breath hold techniques were used in four series (deep inspiration breath hold or DIBH in 3 of them) (12, 15, 19, 29).

**Clinical target volume (CTV)**

The clinical target volume or CTV, which in its strict definition considers a volume of tissue containing subclinical malignant disease, with a certain probability of occurrence relevant to the selected therapy. It may be possible that groups that have used CTV may did so with the aim of considering the uncertainties in target definition. Only five publications provide a definition of the clinical target volume (CTV), specifying in only two the margin used, which was 10 mm in one. In the second publication CTV was identical to the VT substrate.

**Internal target volume (ITV)**

The ITV is usually defined as the CTV plus a safety margin that considers uncertainties in the size, shape and position of the CTV within the patient. Only one group that has defined a CTV used an ITV. In 12 series an internal target volume (ITV) has been reported.

**Planning target volume (PTV)**

The planning target volume or PTV is a geometric concept introduced for treatment planning and evaluation. In the ICRU definition, the PTV is a margin around the CTV, taking into account the patient's internal and setup uncertainties. The delineation of the PTV is technique-dependent and is part of the treatment prescription.

For PTV delineation the use of different scanner and techniques have been reported. In 16 publications a 4D scanner to capture the location of the target over time was used, in addition to a free-breathing CT scanner in 8/16. The advantage of 4D acquisition lies in the fact that the movements, as well as the deformation of the heart and its cavities, are considered at the different moments of the respiratory cycle. This allows calculation of the maximum expansion of the target (through the definition of an ITV in 12 publications) and its geometric relationship with the implanted cardiac defibrillator (ICD) tip in the right ventricle. The latter has been used as a fiducial in most of the patients treated using a CyberKnife system (Accuray Inc., Sunnyvale, CA, USA).

In 13 publications additional image modalities have been used, including CMR in 9 and cardiac PET-CT or cardiac SPECT in 4. Among the 19 publications indicating the use of an electroanatomical mapping (EAM) system, 11 of them have used CARTO (Biosense Webster, Irvine, CA, USA), one used CardioInsight (Medtronic, Minneapolis, MN, USA), and the remaining did not provide details of the system

**Supplemental EAM data for Figure 2 and 6**

EAM data

|  | **Study** | **Patient population (all referred for RFCA of SMVT)** | **BV<1.5mV, cm^2^** | **UV<8.27mV cm^2^** | **Average wall thickness, cm** |
| --- | --- | --- | --- | --- | --- |
| 1 | Kimura et al 2022(36) | 103 DCM patients | 4 [1-17] | 73 [31-118] | 0.95 ± 0.15 |
| 2 | Rademaker et al 2024(37) | 150 ICM patients | 60 [41-86] | 118 [75-180] | N.A. |
| 3 | Tzou et al 2015(38) | 44 ICM and NICM patients | 55 ± 40 | N.A. | N.A. |
| 4 | Berruezo et al 2015(39) | 101 ICM and NICM patients | 52 ± 39 | N.A. | N.A. |

Results are shown as median [IQR] or mean ± standard deviation.
BV, bipolar voltage; UV, unipolar voltage; mV, microvolt; ICM, ischemic cardiomyopathy; CTV, clinical target volume; LV, left ventricle; MI, myocardial infarction; N.A., not available; RAO: right anterior oblique RFCA, radiofrequency catheter ablation; SMVT, sustained monomorphic ventricular tachycardia;

MRI data

|  | **Study** | **Patient population** | **LGE scar mass, g** | **LGE scar core mass, g** | **Total LV mass** |
| --- | --- | --- | --- | --- | --- |
| 1 | Acosta et al 2018(40) | 25 patients with DCM and VT | 38.7 ± 34.2 | 19.0 ± 14.2 | N.A. |
| 2 | Piers et al 2015(41) | 10 patients with DCM and VT | 16.6 [9.5-24.3] | 10.0 [5.6-15.3] | 135 [102–155] |
| 3 | Alexandre et al 2013(42) | 14 patients with ICM and VT | 26.6 ± 14.5 | N.A. | 205 ±63 |
| 4 | Demirel et al 2014(43) | 34 patients with ICM and VT | 52.9 ± 23.7 | 29.7 ± 15.3 | 210.4 ±50.4 |

CardTV and PTV data

|  | **Study** | **Patient population (all referred for STAR of VT)** | **CTV ml** | **PTV, ml** |
| --- | --- | --- | --- | --- |
| 1 | Robinson et al 2018(30) | 11 ICM, 8 NICM patients | 25.4 [6.4-88.6] | 98.9 [60.9-298.8] |
| 2 | Carbucicchio et al 2021(21) | 3 ICM, 4 NICM patients | 39 ± 17 | 185 ± 53 |
| 3 | Chin et al 2020(13) | 4 ICM, 4 NICM patients | 34.2±19.3 | 121 ± 50 |
| 4 | Neuwirth et al 2019(5) | 8 ICM, 2 NICM patients | 22 [14.2 – 29.6] | 22 [14.2 – 29.6] |
| 5 | Siklody et al 2023 (44) | 6 ICM, 14 NICM patients | 16 (range: 4 – 27) | 23 (range: 14 – 36) |
| 6 | Haskova et al 2024 (45) | 5 ICM, 12 NICM patients | 39.4 [22.2 – 62.1] | 39.4 [22.2 – 62.1] |

Results reported as median [IQR] or mean±standard deviation. References for studies in Supplement. DCM, dilated cardiomyopathy; LVEF, left ventricular ejection fraction; LV, left ventricle; LGE, late-gadolinium enhancement; MRI, magnetic resonance imaging; ventricular tachycardia; ICM; ischemic cardiomyopathy; NICM, non-ischemic cardiomyopathy; CTV, clinical target volume; PTV, planning target volume; STAR, stereotactic arrhythmia radioablation; SD, standard deviation; SI, signal intensity

**Supplemental Table 2: Studies reporting on bipolar and unipolar voltage mapping to identify ventricular scar**

| **Bipolar Voltage Mapping** | | | |
| --- | --- | --- | --- |
| **Disease/Chamber** | **Value** | **Population** | **Scar Validation** |
| Normal  Bipolar Voltage(46) | >1.5 mV | 6 patients (5 males, 37±12 years) without structural heart disease: Mapping RV (4 patients) and/or LV (4 patients) | 95% of all RV B-EGMs >1.44 mV.  95% of all LV B-EGMs >1.55 mV. |
| Normal Bipolar Voltage(47) | >1.8 mV | MRI in 15 pts with prior MI | 75^th^ percentile in LV without late gadolinium enhancement (LGE) on CMR |
| Post-MI non-infarcted endocardium(48) | ≥3.0 mV non-remodeled LV  ≥2.1 mv remodeled LV* | 27 consecutive patients (26 male, 65 years) with post-MI VT or PVCs. | Scar validated by LGE-CMR  Low voltage area (infarcted endocardium) contained >97% of targeted evoked delayed potentials. More fibrotic non-infarcted myocardium in patients with remodeled LV compared with non-remodeled LV. |
| Normal epicardium(49) | >1.4  95% of B-EGMs were >1.4 mV (mean 4.0±1.2 mV, range, 0.5 to 19.9 mV) | Porcine model of healed anterior wall myocardial infarction (n=13 animals), | Histology |
| NICM(50) | 2.9 mV differentiated between areas with little (<40 mm2) or greater fibrosis | 8 explanted human hearts prior EAM and MRI imaging | Histology, CMR |
| Dense scar | <0.5 mv bipolar | 158 patients (128 male, 58 ± 10 years) with SHD and endocardial mapping. Non-validated. (51)  14 patients (11 male; age 65.4±11.5 ) with post-MI VT. Scar validated by electrical excitability (threshold >10 mA). (52) | Arbitrary definition of abnormal B-EGMs if <0.5 mV. (51)  98% of LV B-EGMs <0.5 mV in electrically unexcitable (pacing at 10 mA) scar.(52)^8^  24% of LV B-EGMs <0.25 mV unexcitable scar.(52) |
| Dense scar Bipolar (47) | 0.8 mV median (IQR 0.4 – 1.3) mv | 15 pts with prior MI | Transmural scar on LGE-CMR |
| Dense scar Bipolar Voltage (high definition catheters) | <0.76-0.95 mV (depending on the bipole orientation)(53)    <0.2-1.0 mV(54) | 6 infarcted sheep. HD-Grid catheter. Scar validated by CMR.(53)  40 patients (39 males, 67 ± 9 years) post-MI VT. Pentaray Catheter. CT. (54) | LGE-CMR  <0.2 mV, < 0.5 mV and <1 mV best correlated with 2 mm, 3 mm and 5 mm LV wall thinning on CT. |
| LV endocardial VT circuit isthmus (conventional catheter) | 0.26 – 0.32 mV | 24 of 38 consecutive patients (22 male, 66±9) with SHD SMVT. (55)  14 consecutive patients (11 male; age 65.4±11.5 ) with post-MI SMVT.(52) | B-EGMs amplitude is 0.26 ±0.11 mV (range 0.11 to 0.5) during RV pacing. (55)  B-EGMs amplitude is 0.32±0.16 mV (0.08 to 0.91 mV) during sinus rhythm. (52) |
| LV endocardial VT circuit isthmus (high resolution catheters)(56) | 0.06 mV (median) | 31 patients (29 male, 27 post-MI VT) | B-EGMs amplitude is 0.12 mV during tachycardia and 0.06 mV during paced/sinus rhythm. |
| Epicardial VT circuit isthmus (conventional catheter)(57) | 0.39 mV | 22 consecutive patients (20 male, 40±12 years) with ARVC SMVT | 74.7% of conduction channels were found in the epicardial surface. 72.4% were late conduction channels and had 0.39 mV B-EGM amplitude (0.26–0.50) |
| **Unipolar Voltage Mapping** | | | |
| **Disease/Chamber** | **Value** | **Population** | **Scar Validation** |
| Normal LV(58, 59) | >8.3  95% of all LV U-EGMs >8.27 mV. (mean, 19.6±6.9 mV) | 6 patients (5 males, 36±18 years) without structural heart disease. | No heart disease |
| Post-MI non-infarcted endocardium(48) | ≥6.7 non-remodeled LV  ≥6.4 remodeled LV* | 27 consecutive patients (26 male, 65 years) with post-MI VT or PVCs. | Scar validated by LGE-CMR |
| LV epicardial scar  Unipolar endocardial Voltage(60) | <5.1 mV | 31 patients with scar-related VT (Post-MI, CAD, Sarcoid, NICM) | LV U-EGMs <5.1 mV (sensitivity 91%, specificity 75%) for predicting epicardial scar from bipolar epicardial mapping. |
| NICM LV(50) | 2.9 mV (bipolar) and 6.85 mV (unipolar) differentiated between areas with little (<40 mm2) or greater fibrosis | 8 explanted human hearts with NICM and prior EAM and MRI imaging | Uni and Bipolar voltage linearly related to fibrosis.  Histology |
| Normal RV(61) | >5.5  95% of all RV U-EGMs >5.5 mV | 8 patients (6 males, 36 ± 18 years) without structural heart disease |  |
| Normal RV (anterior septum)  Unipolar Voltage(62) | 7.5 mV | 9 patients (5 males; 49 ± 11 years) without structural heart disease; | U-EGMs in the posterior region of the septal aspect of the RV have lower amplitude (mean 6.56 ± 2.33 mV, 95% CI 6.08–7.05), compared to the anterior region (mean 8.33 ± 2.34 mV, P < 0.001, 95% CI 7.848.84). |
| Normal RV (posterior septum)  Unipolar Voltage(62) | 6.0 mV | 9 patients (5 males; 49 ± 11 years) without structural heart disease; |  |
| RV epicardial scar  Unipolar Voltage(60) | <4.4 mV  RV U-EGMs <4.4 mV (sensitivity 93%, specificity 76%) for predicting epicardial scar | 31 patients with scar-related ventricular tachycardia (Post-MI, CAD, Sarcoid, NICM) | Epicardial electroanatomic mapping. |
| RV epicardial scar independent of epicardial fat  Unipolar Voltage(63) | <3.9 mV | 33 patients (26 males, 50±14 years, body mass index 25±4 kg/m2) and RV scar–related VT. | Epicardial low voltage (<1.5 mV) B-EGMs |

**: defined by LV end-systolic volume index of >50 ml/m2 and ejection fraction of <47%. ARVC, arrhythmogenic ventricular cardiomyopathy; B-EGMs, bipolar electrograms; CMR, Cardiac Magnetic Resonance; CT, Computed Tomography; EGMs, electrograms, LV, left ventricle; MI, myocardial infarction; PVC, premature ventricular contraction; RV, Right ventricle; SHD, structural heart disease; U-EGMs, unipolar electrograms; VT, ventricular tachycardia*

**Supplemental Table 3: Compilation of results validating ECGI against contact data.**

| **Study** | **Subjects** | **EGM Correlation** | **AT** | **RT** | **Localisation Accuracy (mm)** | **Type** |
| --- | --- | --- | --- | --- | --- | --- |
| Oster et al. (1997)(64) | 1 | 0.9 | N/A | N/A | <10 | Torso-Tank |
| Burnes et al (2001)(65) | 1 | 0.90 | N/A | N/A | 8.69 | Torso-Tank |
| Bear et al. (2018)(66) | 8 | N/A | R = 0.68 | N/A | 9.1 | Torso-Tank |
| Bear et al. (2019)(67) | 3 | N/A | 0.79 | N/A | N/A | Torso-Tank |
| Cluitman et al. (2017)(68) | 4 | 0.71 | 0.82 | 0.73 | 10 | Canine |
| Bear et al. (2018)(66) | 5 | 0.72 | 0.72 | N/A | 16 | Porcine |
| Ghanem et al. (2004)(69) | 3 | 0.72 | N/A | N/A | 13 | Human |
| Sapp et al. (2012)(70) | 4 | N/A | N/A | N/A | 13 | Human |

References

1. cvek J, Neuwirth R, Knybel L, Molenda L, Otahal L, Pindor J, Murarova M, Kodaj M, Fiala M, Branny M, Feltl D. Cardiac radiosurgery for malignant ventricular tachycardia. Cureus. 2014;6(7):1-7.

2. Loo BW, Jr., Soltys SG, Wang L, Lo A, Fahimian BP, Iagaru A, et al. Stereotactic ablative radiotherapy for the treatment of refractory cardiac ventricular arrhythmia. Circulation Arrhythmia and electrophysiology. 2015;8(3):748-50.

3. Jumeau R, Ozsahin M, Schwitter J, Vallet V, Duclos F, Zeverino M, et al. Rescue procedure for an electrical storm using robotic non-invasive cardiac radio-ablation. Radiother Oncol. 2018;128(2):189-91.

4. Haskova J, Peichl P, Pirk J, Cvek J, Neuwirth R, Kautzner J. Stereotactic radiosurgery as a treatment for recurrent ventricular tachycardia associated with cardiac fibroma. HeartRhythm Case Rep. 2019;5(1):44-7.

5. Neuwirth R, Cvek J, Knybel L, Jiravsky O, Molenda L, Kodaj M, et al. Stereotactic radiosurgery for ablation of ventricular tachycardia. EP Europace. 2019;21:1088-95.

6. Zeng LJ, Huang LH, Tan H, Zhang HC, Mei J, Shi HF, et al. Stereotactic body radiation therapy for refractory ventricular tachycardia secondary to cardiac lipoma: A case report. Pacing Clin Electrophysiol. 2019;42(9):1276-9.

7. Gianni C, Rivera D, Burkhardt JD, Pollard B, Gardner E, Maguire P, et al. Stereotactic arrhythmia radioablation for refractory scar-related ventricular tachycardia. Heart Rhythm. 2020;17(8):1241-8.

8. Haskova J, Jedlickova K, Cvek J, Knybel L, Neuwirth R, Kautzner J. Oesophagopericardial fistula as a late complication of stereotactic radiotherapy for recurrent ventricular tachycardia. Europace. 2022;24(6):969.

9. Ninni S, Gallot-Lavallee T, Klein C, Longere B, Brigadeau F, Potelle C, et al. Stereotactic Radioablation for Ventricular Tachycardia in the Setting of Electrical Storm. Circulation Arrhythmia and electrophysiology. 2022;15(9):e010955.

10. Qian PC, Quadros K, Aguilar M, Wei C, Boeck M, Bredfeldt J, et al. Substrate Modification Using Stereotactic Radioablation to Treat Refractory Ventricular Tachycardia in Patients With Ischemic Cardiomyopathy. JACC Clin Electrophysiol. 2022;8(1):49-58.

11. Carbucicchio C, Jereczek-Fossa BA, Andreini D, Catto V, Piperno G, Conte E, et al. STRA-MI-VT (STereotactic RadioAblation by Multimodal Imaging for Ventricular Tachycardia): rationale and design of an Italian experimental prospective study. J Interv Card Electrophysiol. 2021;61(3):583-93.

12. Cha MJ, Cuculich PS, Robinson CG, Chang JH. Tailored stereotactic radiotherapy technique using deep inspiration breath-hold to reduce stomach dose for cardiac radioablation. Radiat Oncol J. 2021;39(3):167-73.

13. Chin R, Hayase J, Hu P, Cao M, Deng J, Ajijola O, et al. Non-invasive stereotactic body radiation therapy for refractory ventricular arrhythmias: an institutional experience. J Interv Card Electrophysiol. 2021;61(3):535-43.

14. Chiu MH, Mitchell LB, Ploquin N, Faruqi S, Kuriachan VP. Review of Stereotactic Arrhythmia Radioablation Therapy for Cardiac Tachydysrhythmias. CJC Open. 2021;3(3):236-47.

15. Cybulska M, Sajdok M, Bednarek J, Miszczyk M, Jadczyk T, Kurzelowski R, et al. Stereotactic arrhythmia radioablation in recurrent ventricular tachyarrhythmias. Kardiol Pol. 2022;80(3):367-9.

16. Knutson NC, Samson PP, Hugo GD, Goddu SM, Reynoso FJ, Kavanaugh JA, et al. Radiation Therapy Workflow and Dosimetric Analysis from a Phase 1/2 Trial of Noninvasive Cardiac Radioablation for Ventricular Tachycardia. Int J Radiat Oncol Biol Phys. 2019;104(5):1114-23.

17. Lee J, Bates M, Shepherd E, Riley S, Henshaw M, Metherall P, et al. Cardiac stereotactic ablative radiotherapy for control of refractory ventricular tachycardia: initial UK multicentre experience. Open Heart. 2021;8(2).

18. Levis M, Dusi V, Magnano M, Cerrato M, Gallio E, Depaoli A, et al. A case report of long-term successful stereotactic arrhythmia radioablation in a cardiac contractility modulation device carrier with giant left atrium, including a detailed dosimetric analysis. Front Cardiovasc Med. 2022;9:934686.

19. Narducci ML, Cellini F, Placidi L, Boldrini L, Perna F, Bencardino G, et al. Case Report: A Case Report of Stereotactic Ventricular Arrhythmia Radioablation (STAR) on Large Cardiac Target Volume by Highly Personalized Inter- and Intra-fractional Image Guidance. Front Cardiovasc Med. 2020;7:565471.

20. van der Ree MH, Dieleman EMT, Visser J, Adam JA, de Bruin-Bon RHA, de Jong R, et al. Direct Clinical Effects of Cardiac Radioablation in the Treatment of a Patient With Therapy-Refractory Ventricular Tachycardia Storm. Adv Radiat Oncol. 2022;7(5):100992.

21. Carbucicchio C, Andreini D, Piperno G, Catto V, Conte E, Cattani F, et al. Stereotactic radioablation for the treatment of ventricular tachycardia: preliminary data and insights from the STRA-MI-VT phase Ib/II study. J Interv Card Electrophysiol. 2021;62(2):427-39.

22. Lee Y, Yoon HI, Kim JS, Kim AY, Tsevendee S, Uhm JS. Incessant ventricular tachycardia treated with cardiac radioablation in an 11-year-old boy with dilated cardiomyopathy. HeartRhythm Case Rep. 2021;7(3):186-90.

23. Mayinger M, Kovacs B, Tanadini-Lang S, Ehrbar S, Wilke L, Chamberlain M, et al. First magnetic resonance imaging-guided cardiac radioablation of sustained ventricular tachycardia. Radiother Oncol. 2020;152:203-7.

24. Aras D, Cetin EHO, Ozturk HF, Ozdemir E, Kara M, Ekizler FA, et al. Stereotactic body radioablation therapy as an immediate and early term antiarrhythmic palliative therapeutic choice in patients with refractory ventricular tachycardia. J Interv Card Electrophysiol. 2022.

25. Blanck O, Buergy D, Vens M, Eidinger L, Zaman A, Krug D, et al. Radiosurgery for ventricular tachycardia: preclinical and clinical evidence and study design for a German multi-center multi-platform feasibility trial (RAVENTA). Clin Res Cardiol. 2020;109(11):1319-32.

26. Cuculich PS, Schill MR, Kashani R, Mutic S, Lang A, Cooper D, et al. Noninvasive cardiac radiation for ablation of ventricular tachycardia. New England Journal of Medicine. 2017;377:2325-36.

27. Krug D, Blanck O, Demming T, Dottermusch M, Koch K, Hirt M, et al. Stereotactic body radiotherapy for ventricular tachycardia (cardiac radiosurgery) : First-in-patient treatment in Germany. Strahlenther Onkol. 2020;196(1):23-30.

28. Marti-Almor J, Jimenez-Lopez J, Rodriguez de Dios N, Tizon H, Valles E, Algara M. Noninvasive ablation of ventricular tachycardia with stereotactic radiotherapy in a patient with arrhythmogenic right ventricular cardiomyopathy. Rev Esp Cardiol (Engl Ed). 2020;73(1):97-9.

29. Park JS, Choi Y. Stereotactic Cardiac Radiation to Control Ventricular Tachycardia and Fibrillation Storm in a Patient with Apical Hypertrophic Cardiomyopathy at Burnout Stage: Case Report. J Korean Med Sci. 2020;35(27):e200.

30. Robinson CG, Samson PP, Moore KMS, Hugo GD, Knutson N, Mutic S, et al. Phase I/II trial of electrophysiology-guided noninvasive cardiac radioablation for ventricular tachycardia. Circulation. 2019;139:313-21.

31. Bhaskaran A, Downar E, Chauhan VS, Lindsay P, Nair K, Ha A, et al. Electroanatomical mapping-guided stereotactic radiotherapy for right ventricular tachycardia storm. HeartRhythm Case Rep. 2019;5(12):590-2.

32. Krug D, Blanck O, Andratschke N, Guckenberger M, Jumeau R, Mehrhof F, et al. Recommendations regarding cardiac stereotactic body radiotherapy for treatment refractory ventricular tachycardia. Heart Rhythm. 2021;18(12):2137-45.

33. Lloyd MS, Wight J, Schneider F, Hoskins M, Attia T, Escott C, et al. Clinical experience of stereotactic body radiation for refractory ventricular tachycardia in advanced heart failure patients. Heart Rhythm. 2020;17(3):415-22.

34. Robinson CG, Samson PP, Moore KMS, Hugo GD, Knutson N, Mutic S, et al. Phase I/II Trial of Electrophysiology-Guided Noninvasive Cardiac Radioablation for Ventricular Tachycardia. Circulation. 2019;139(3):313-21.

35. Scholz EP, Seidensaal K, Naumann P, Andre F, Katus HA, Debus J. Risen from the dead: Cardiac stereotactic ablative radiotherapy as last rescue in a patient with refractory ventricular fibrillation storm. HeartRhythm Case Rep. 2019;5(6):329-32.

36. Kimura Y, Beukers HKC, Rademaker R, Chen HS, Ebert M, Jensen T, et al. Volume-Weighted Unipolar Voltage Predicts Heart Failure Mortality in Patients With Dilated Cardiomyopathy and Ventricular Arrhythmias. JACC Clin Electrophysiol. 2022.

37. Rademaker R, Kimura Y, de Riva Silva M, Beukers HC, Piers SRD, Wijnmaalen AP, et al. Area-weighted unipolar voltage to predict heart failure outcomes in patients with ischaemic cardiomyopathy and ventricular tachycardia. Europace. 2024;26(2).

38. Tzou WS, Frankel DS, Hegeman T, Supple GE, Garcia FC, Santangeli P, et al. Core isolation of critical arrhythmia elements for treatment of multiple scar-based ventricular tachycardias. Circulation Arrhythmia and electrophysiology. 2015;8(2):353-61.

39. Berruezo A, Fernandez-Armenta J, Andreu D, Penela D, Herczku C, Evertz R, et al. Scar dechanneling: new method for scar-related left ventricular tachycardia substrate ablation. Circulation Arrhythmia and electrophysiology. 2015;8(2):326-36.

40. Acosta J, Fernandez-Armenta J, Borras R, Anguera I, Bisbal F, Marti-Almor J, et al. Scar Characterization to Predict Life-Threatening Arrhythmic Events and Sudden Cardiac Death in Patients With Cardiac Resynchronization Therapy: The GAUDI-CRT Study. JACC Cardiovasc Imaging. 2018;11(4):561-72.

41. Piers SR, Everaerts K, van der Geest RJ, Hazebroek MR, Siebelink HM, Pison LA, et al. Myocardial scar predicts monomorphic ventricular tachycardia but not polymorphic ventricular tachycardia or ventricular fibrillation in nonischemic dilated cardiomyopathy. Heart Rhythm. 2015;12(10):2106-14.

42. Alexandre J, Saloux E, Dugue AE, Lebon A, Lemaitre A, Roule V, et al. Scar extent evaluated by late gadolinium enhancement CMR: a powerful predictor of long term appropriate ICD therapy in patients with coronary artery disease. J Cardiovasc Magn Reson. 2013;15(1):12.

43. Demirel F, Adiyaman A, Timmer JR, Dambrink JH, Kok M, Boeve WJ, et al. Myocardial scar characteristics based on cardiac magnetic resonance imaging is associated with ventricular tachyarrhythmia in patients with ischemic cardiomyopathy. Int J Cardiol. 2014;177(2):392-9.

44. Herrera Siklody C, Schiappacasse L, Jumeau R, Reichlin T, Saguner AM, Andratschke N, et al. Recurrences of ventricular tachycardia after stereotactic arrhythmia radioablation arise outside the treated volume: analysis of the Swiss cohort. Europace. 2023;25(10).

45. Haskova J, Wichterle D, Kautzner J, Sramko M, Peichl P, Knybel PL, et al. Efficacy and Safety of Stereotactic Radiotherapy in Patients With Recurrent Ventricular Tachycardias: The Czech Experience. JACC Clin Electrophysiol. 2024.

46. Marchlinski FE, Callans DJ, Gottlieb CD, Zado E. Linear ablation lesions for control of unmappable ventricular tachycardia in patients with ischemic and nonischemic cardiomyopathy. Circulation. 2000;101(11):1288-96.

47. Wijnmaalen AP, van der Geest RJ, van Huls van Taxis CF, Siebelink HM, Kroft LJ, Bax JJ, et al. Head-to-head comparison of contrast-enhanced magnetic resonance imaging and electroanatomical voltage mapping to assess post-infarct scar characteristics in patients with ventricular tachycardias: real-time image integration and reversed registration. Eur Heart J. 2011;32(1):104-14.

48. Sramko M, Abdel-Kafi S, van der Geest RJ, de Riva M, Glashan CA, Lamb HJ, et al. New Adjusted Cutoffs for "Normal" Endocardial Voltages in Patients With Post-Infarct LV Remodeling. JACC Clin Electrophysiol. 2019;5(10):1115-26.

49. Reddy VY, Wrobleski D, Houghtaling C, Josephson ME, Ruskin JN. Combined epicardial and endocardial electroanatomic mapping in a porcine model of healed myocardial infarction. Circulation. 2003;107(25):3236-42.

50. Glashan CA, Androulakis AFA, Tao Q, Glashan RN, Wisse LJ, Ebert M, et al. Whole human heart histology to validate electroanatomical voltage mapping in patients with non-ischaemic cardiomyopathy and ventricular tachycardia. Eur Heart J. 2018;39(31):2867-75.

51. Cassidy DM, Vassallo JA, Miller JM, Poll DS, Buxton AE, Marchlinski FE, et al. Endocardial catheter mapping in patients in sinus rhythm: relationship to underlying heart disease and ventricular arrhythmias. Circulation. 1986;73(4):645-52.

52. Soejima K, Stevenson WG, Maisel WH, Sapp JL, Epstein LM. Electrically unexcitable scar mapping based on pacing threshold for identification of the reentry circuit isthmus: feasibility for guiding ventricular tachycardia ablation. Circulation. 2002;106(13):1678-83.

53. Takigawa M, Relan J, Kitamura T, Martin CA, Kim S, Martin R, et al. Impact of Spacing and Orientation on the Scar Threshold With a High-Density Grid Catheter. Circulation Arrhythmia and electrophysiology. 2019;12(9):e007158.

54. Ene E, Halbfass P, Nentwich K, Sonne K, Berkovitz A, Cochet H, et al. Optimal cut-off value for endocardial bipolar voltage mapping using a multipoint mapping catheter to characterize the scar regions described in cardio-CT with myocardial thinning. Journal of cardiovascular electrophysiology. 2022;33(10):2174-80.

55. Arenal A, Glez-Torrecilla E, Ortiz M, Villacastin J, Fdez-Portales J, Sousa E, et al. Ablation of electrograms with an isolated, delayed component as treatment of unmappable monomorphic ventricular tachycardias in patients with structural heart disease. J Am Coll Cardiol. 2003;41(1):81-92.

56. Martin R, Maury P, Bisceglia C, Wong T, Estner H, Meyer C, et al. Characteristics of Scar-Related Ventricular Tachycardia Circuits Using Ultra-High-Density Mapping: A Multi-Center Study. Circulation Arrhythmia and electrophysiology. 2018;11(10):e006569.

57. Fernandez-Armenta J, Andreu D, Penela D, Trucco E, Cipolletta L, Arbelo E, et al. Sinus rhythm detection of conducting channels and ventricular tachycardia isthmus in arrhythmogenic right ventricular cardiomyopathy. Heart Rhythm. 2014;11(5):747-54.

58. Hutchinson MD, Gerstenfeld EP, Desjardins B, Bala R, Riley MP, Garcia FC, et al. Endocardial unipolar voltage mapping to detect epicardial ventricular tachycardia substrate in patients with nonischemic left ventricular cardiomyopathy. Circulation Arrhythmia and electrophysiology. 2011;4(1):49-55.

59. Campos FO, Orini M, Arnold R, Whitaker J, O'Neill M, Razavi R, et al. Assessing the ability of substrate mapping techniques to guide ventricular tachycardia ablation using computational modelling. Comput Biol Med. 2021;130:104214.

60. Tokuda M, Tedrow UB, Inada K, Reichlin T, Michaud GF, John RM, et al. Direct comparison of adjacent endocardial and epicardial electrograms: implications for substrate mapping. J Am Heart Assoc. 2013;2(5):e000215.

61. Polin GM, Haqqani H, Tzou W, Hutchinson MD, Garcia FC, Callans DJ, et al. Endocardial unipolar voltage mapping to identify epicardial substrate in arrhythmogenic right ventricular cardiomyopathy/dysplasia. Heart Rhythm. 2011;8(1):76-83.

62. Kelesidis I, Desjardins B, Muser D, Santangeli P, Zado ES, Marchlinski FE. Unipolar voltage mapping criteria for right ventricular septum: Influence of the aortic root. Journal of cardiovascular electrophysiology. 2018;29(8):1113-8.

63. Venlet J, Piers SRD, Kapel GFL, de Riva M, Pauli PFG, van der Geest RJ, et al. Unipolar Endocardial Voltage Mapping in the Right Ventricle: Optimal Cutoff Values Correcting for Computed Tomography-Derived Epicardial Fat Thickness and Their Clinical Value for Substrate Delineation. Circulation Arrhythmia and electrophysiology. 2017;10(8).

64. Oster HS, Taccardi B, Lux RL, Ershler PR, Rudy Y. Noninvasive electrocardiographic imaging: reconstruction of epicardial potentials, electrograms, and isochrones and localization of single and multiple electrocardiac events. Circulation. 1997;96(3):1012-24.

65. Burnes JE, Taccardi B, Ershler PR, Rudy Y. Noninvasive electrocardiogram imaging of substrate and intramural ventricular tachycardia in infarcted hearts. J Am Coll Cardiol. 2001;38(7):2071-8.

66. Bear LR, Huntjens PR, Walton RD, Bernus O, Coronel R, Dubois R. Cardiac electrical dyssynchrony is accurately detected by noninvasive electrocardiographic imaging. Heart Rhythm. 2018;15(7):1058-69.

67. Bear LR, Walton RD, Abell E, Coudiere Y, Haissaguerre M, Bernus O, et al. Optical Imaging of Ventricular Action Potentials in a Torso Tank: A New Platform for Non-Invasive Electrocardiographic Imaging Validation. Front Physiol. 2019;10:146.

68. Cluitmans MJM, Bonizzi P, Karel JMH, Das M, Kietselaer B, de Jong MMJ, et al. In Vivo Validation of Electrocardiographic Imaging. JACC Clin Electrophysiol. 2017;3(3):232-42.

69. Ghanem RN, Jia P, Ramanathan C, Ryu K, Markowitz A, Rudy Y. Noninvasive electrocardiographic imaging (ECGI): comparison to intraoperative mapping in patients. Heart Rhythm. 2005;2(4):339-54.

70. Sapp JL, Dawoud F, Clements JC, Horacek BM. Inverse solution mapping of epicardial potentials: quantitative comparison with epicardial contact mapping. Circulation Arrhythmia and electrophysiology. 2012;5(5):1001-9.
